# Dysregulation of the progranulin-driven autophagy-lysosomal pathway mediates secretion of the nuclear protein TDP-43

**DOI:** 10.1016/j.jbc.2023.105272

**Published:** 2023-09-20

**Authors:** Yoshinori Tanaka, Shun-ichi Ito, Yuki Honma, Masato Hasegawa, Fuyuki Kametani, Genjiro Suzuki, Lina Kozuma, Kosuke Takeya, Masumi Eto

**Affiliations:** 1 Biochemistry Unit, Faculty of Veterinary Medicine, Okayama University of Science, Imabari-shi, Ehime, Japan; 2 Department of Brain and Neurosciences, Tokyo Metropolitan Institute of Medical Science, Tokyo, Japan

**Keywords:** autophagy, extracellular vesicles, TAR DNA-binding protein 43, frontotemporal lobar degeneration, amyotrophic lateral sclerosis, progranulin, lysosome, autophagosome, autolysosome

## Abstract

The cytoplasmic accumulation of the nuclear protein transactive response DNA-binding protein 43 kDa (TDP-43) has been linked to the progression of amyotrophic lateral sclerosis and frontotemporal lobar degeneration. TDP-43 secreted into the extracellular space has been suggested to contribute to the cell-to-cell spread of the cytoplasmic accumulation of TDP-43 throughout the brain; however, the underlying mechanisms remain unknown. We herein demonstrated that the secretion of TDP-43 was stimulated by the inhibition of the autophagy-lysosomal pathway driven by progranulin (PGRN), a causal protein of frontotemporal lobar degeneration. Among modulators of autophagy, only vacuolar-ATPase inhibitors, such as bafilomycin A1 (Baf), increased the levels of the full-length and cleaved forms of TDP-43 and the autophagosome marker LC3-II (microtubule-associated proteins 1A/1B light chain 3B) in extracellular vesicle fractions prepared from the culture media of HeLa, SH-SY5Y, or NSC-34 cells, whereas vacuolin-1, MG132, chloroquine, rapamycin, and serum starvation did not. The C-terminal fragment of TDP-43 was required for Baf-induced TDP-43 secretion. The Baf treatment induced the translocation of the aggregate-prone GFP-tagged C-terminal fragment of TDP-43 and mCherry-tagged LC3 to the plasma membrane. The Baf-induced secretion of TDP-43 was attenuated in autophagy-deficient ATG16L1 knockout HeLa cells. The knockdown of PGRN induced the secretion of cleaved TDP-43 in an autophagy-dependent manner in HeLa cells. The KO of PGRN in mouse embryonic fibroblasts increased the secretion of the cleaved forms of TDP-43 and LC3-II. The treatment inducing TDP-43 secretion increased the nuclear translocation of GFP-tagged transcription factor EB, a master regulator of the autophagy-lysosomal pathway in SH-SY5Y cells. These results suggest that the secretion of TDP-43 is promoted by dysregulation of the PGRN-driven autophagy-lysosomal pathway.

The abnormal aggregation of the nuclear protein, transactive response DNA-binding protein 43 kDa (TDP-43) in the cytoplasm is a hallmark of amyotrophic lateral sclerosis (ALS) and frontotemporal lobar degeneration (FTLD-TDP) (1, 2). TDP-43 is a ubiquitously expressed RNA-binding protein (3). Its pathological accumulation in the cytoplasm causes the loss of function of TDP-43 in the nucleus and the toxic gain of function of TDP-43 in the cytoplasm (4). Pathological TDP-43 is detergent insoluble, hyperphosphorylated, ubiquitinated, and abnormally cleaved to generate aggregation-prone C-terminal fragments (5, 6, 7, 8). In affected regions in ALS and FTLD-TDP patients, C-terminal fragments of TDP-43 form a fibril-like structure, which comprises a single protofilament. The low-complexity domain of C-terminal TDP-43 (amino acids 282–360) is a key structural element of the protofilament (9). A detergent-insoluble fraction containing TDP-43 fibrils prepared from ALS and FTLD-TDP brains is capable of inducing conformational changes in normal TDP-43 to the pathological form, which accelerates the accumulation of TDP-43 (8). Moreover, most of the TDP-43 mutations identified in familial and sporadic ALS are concentrated within the C-terminal region, a known aggregate-prone domain (10). Therefore, the mechanisms by which C-terminal fragments of TDP-43 are formed and propagate need to be elucidated in order to obtain a more detailed understanding of the roles of TDP-43 underlying ALS/FTLD-TDP.

The pathology of cytoplasmic accumulation of TDP-43 spreads through the brain and spinal cord with the progression of ALS and FTLD-TDP and is closely involved in the symptoms of these diseases (11). TDP-43 is detected in the cerebrospinal fluid (CSF), and elevated levels of TDP-43 have been found in the CSF of ALS and FTLD-TDP patients (12, 13). TDP-43 is secreted through extracellular vesicles (EVs) with lipid bilayer membranes and is taken up and transmitted by axon terminals (14). Furthermore, an intracerebral injection of the detergent-insoluble fraction prepared from the brain of an FTLD-TDP patient induced the *de novo* pathology of cytoplasmic accumulation of TDP-43 in a mouse brain, which progressively spread throughout the brain in a time-dependent manner *via* the neuroanatomic connectome (15). These findings suggest that TDP-43 secreted *via* vesicles triggers the pathological formation and cell-to-cell spread of TDP-43 aggregates. However, the mechanisms by which TDP-43 aggregates are secreted into the extracellular space *via* EV remain unclear.

Autophagy is a highly conserved cellular degradation process that involves autophagosomes, which are cytosolic double-membraned vesicles. The formation of autophagosomes begins with a membrane cistern called a phagophore, which grows around a specific region of the cytoplasm and isolates an unnecessary area within the autophagosome. The organelle then fuses with lysosomes to form autolysosomes for the degradation of its contents (16, 17, 18, 19). In the sporadic ALS patients, autophagic structures engulfing cytoplasmic contents such as a skein-like inclusion are enriched in the cytoplasm of neurons, suggesting that autolysosome formation is inhibited (20). To support this idea, causal genes of familial ALS have been suggested to regulate the autophagy-lysosome pathway (21, 22). The previous study indicated that prevention of autophagy-lysosome pathway increased secretion of autophagic components and autophagic cargo receptors *via* EV (23). The secretion of HMGB1 (high mobility group box 1), a nuclear protein incorporated into autophagosomes, was previously shown to be inhibited by wortmannin, an inhibitor of autophagosome formation, and was reduced in autophagy-deficient ATG5 KO mouse embryonic fibroblasts (MEFs) but was enhanced by bafilomycin A1 (Baf), an inhibitor of vacuolar-ATPase (v-ATPase) and autolysosome formation (24), suggesting the augmented secretion of HMGB1 by inhibition of the autophagy-lysosome pathway. Importantly, TDP-43 has been detected in EV with a single- or double-layered membrane, such as exosomes and autophagosomes (14, 25, 26). TDP-43 inclusions localize to endosomes and lysosomes (27) and are degraded by autophagy (28, 29, 30, 31). These findings suggest that aggregate-prone TDP-43 is sequestered by autophagosomes. Nevertheless, it currently remains unclear whether the prevention of autophagosome–lysosome fusion leads to the pathological sequestration of TDP-43 within autophagosomes and its subsequent release into the extracellular environment.

A progranulin (PGRN) haploinsufficiency because of a loss-of-function mutation in the PGRN gene causes FTLD-TDP (32, 33). Although the function of PGRN remains debatable (34), evidence suggests that it controls lysosomal functions (35, 36, 37, 38, 39) and autophagy (29, 36, 40, 41, 42). High numbers of EV were detected in PGRN-deficient mice and FTLD-TDP patients with a PGRN gene mutation (43). Therefore, insufficient PGRN may result in the dysregulation of the autophagy-lysosomal pathway, which, in turn, contributes to the intercellular transmission of pathological TDP-43 aggregates through EV.

In the present study, we aimed to examine whether the autophagy-lysosome pathway regulates extracellular release of TDP-43 *via* EV. To elucidate the mechanisms underlying the release of TDP-43 into the extracellular space and the role of PGRN in this process, we examined the secretion of TDP-43 using different classes of modulators for autophagy. We used the v-ATPase inhibitors, Baf and concanamycin A (Con), the PIKfyve (phosphoinositide kinase, FYVE-type zinc finger containing) inhibitor, vacuolin-1 (Vac), and the autophagosome–lysosome fusion inhibitor, chloroquine (CQ), the autophagy inducer, rapamycin (Rapa), and serum starvation (SS) as well as the knockdown of siRNA.

## Results

### The Baf treatment increases TDP-43 and LC3-II levels in EV fractions

**Figure 1:**
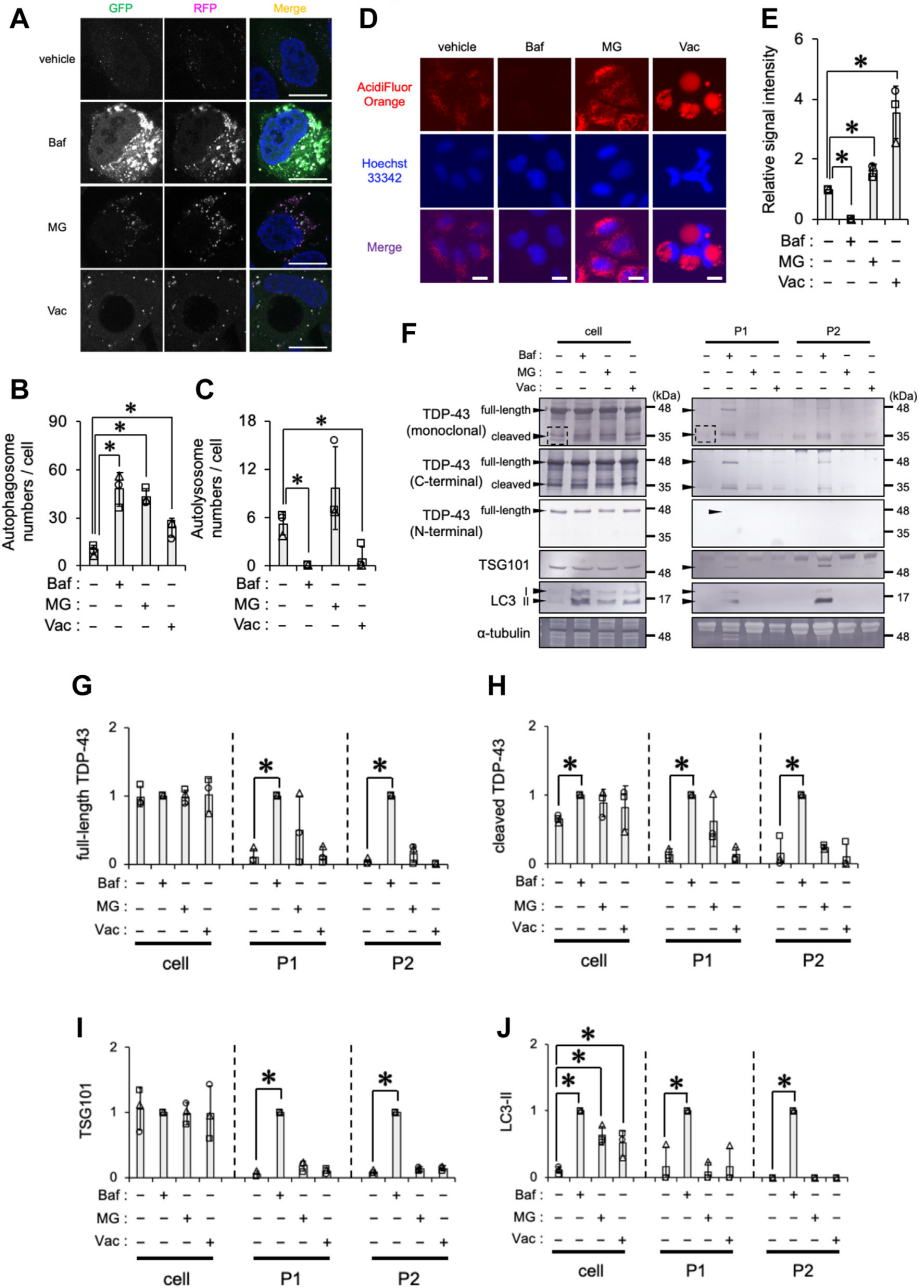
**Bafilomycin A1 (Baf) increases TDP-43, TSG101, and LC3-II levels in the EV fraction.***A*–*C*, HeLa cells expressing mRFP-GFP-LC3 were exposed to vehicle (DMSO), 100 nM Baf, 1 μM MG, or 100 nM Vac for 24 h. *A*, representative images of these cells are shown. Nuclei were stained with DAPI (*blue*). Scale bar represents 20 μm. *B*, autophagosome and (*C*) autolysosome numbers calculated from three independent experiments including at least 20 cells are shown. *D* and *E*, HeLa cells exposed to vehicle (DMSO), 100 nM Baf, 1 μM MG, or 100 nM Vac for 24 h were stained with AcidiFluor ORANGE. *D*, representative images of live cells stained with AcidiFluor ORANGE (*red*) in each treatment. Nuclei were stained with Hoechst 33342 (*blue*). Scale bar represents 20 μm. *E*, relative signal intensities normalized against cells exposed to vehicle were calculated from three independent experiments including at least 330 cells. *F*–*J*, HeLa cells were exposed to DMSO, 100 nM Baf, 1 μM MG, or 100 nM Vac for 24 h. The cell, P1 (20,000*g* pellet), and P2 (110,000*g* pellet) fractions were prepared as shown in the Experimental procedures section. *F*, representative immunoblots (TDP-43 detected by monoclonal, C-terminal, and N-terminal antibodies, TSG101, LC3, and α-tubulin) of each fraction are shown. The *dashed square* indicates the bands of cleaved TDP-43 used for quantification. *G*–*J*, densitometric data on (*G*) full-length TDP-43, (*H*) cleaved TDP-43, (*I*) TSG101, and (*J*) LC3-II in the cell, P1, and P2 fractions were calculated from immunoblotting results. Relative signal intensities normalized against cells exposed to Baf are shown. In bar graphs, data are presented as means ± SD (N = 3). ∗ indicates *p* < 0.05 by the two-tailed unpaired *t* test or Mann–Whitney *U* test. DAPI, 4′,6-diamidino-2-phenylindole; DMSO, dimethyl sulfoxide; EV, extracellular vesicle; LC3, microtubule-associated proteins 1A/1B light chain 3B; MG, MG132; mRFP, monomeric red fluorescent protein; TDP-43, TAR DNA-binding protein 43 kDa; TSG101, tumor susceptibility gene 101 protein; Vac, vacuolin-1.

To elucidate the relationship between TDP-43 secretion and autophagy, we investigated whether stimulators of TDP-43 secretion, such as Baf, MG132 (MG), and Vac (26, 44), induced the formation of autophagosomes and autolysosomes in HeLa cells expressing monomeric red fluorescent protein (mRFP)-GFP-LC3 (microtubule-associated proteins 1A/1B light chain 3B) (Fig. 1*A*). HeLa cells expressing the fluorescence autophagy marker were treated with 100 nM Baf, an inhibitor of v-ATPase and autophagosome–lysosome fusion, 1 μM MG, an inhibitor of proteasome degradation and an inducer of autophagy, or 100 nM Vac, an inhibitor of PIKfyve and autophagosome–lysosome fusion, for 24 h (45, 46) (Fig. 1*A*). LC3-II-positive autophagosomes were identified as yellow puncta because of merged GFP and RFP signals and autolysosomes as red puncta because of the loss of the GFP signal under low pH (or acidic) conditions. Baf, MG, and Vac significantly increased yellow puncta, indicating a higher number of autophagosomes (Fig. 1*B*). On the other hand, the formation of autolysosomes was eliminated by Baf and Vac but not by MG (Fig. 1*C*). These results suggest that the increase in the number of autophagosomes induced by Baf and Vac was due to the suppression of autophagosome–lysosome fusion, whereas MG accelerated the formation of autophagosomes. Autophagic flux was assessed using HeLa cells stably expressing GFP-LC3-RFP-LC3ΔG (Fig. S1). GFP-LC3-RFP-LC3ΔG expressed in cells was previously shown to be cleaved into the autophagy marker GFP-LC3 and the internal control RFP-LC3ΔG at a ratio of 1:1 (45). Therefore, an increase in the GFP/RFP ratio indicates the suppression of autophagic flux. Baf increased the GFP/RFP ratio, suggesting its suppression of autophagic flux (Fig. S1, *A* and *B*). On the other hand, MG decreased the GFP/RFP ratio, indicating its facilitation of autophagic flux (Fig. S1, *C* and *D*). The GFP/RFP ratio of Vac-treated cells was similar to that of vehicle-treated cells (Fig. S1, *C* and *D*). Immunostaining of the phagophore marker WIPI2 (WD repeat domain phosphoinositide interacting 2) showed a significant increase in signal intensity with both Baf and MG but not with Vac (Fig. S2, *A* and *B*). These results suggest that Baf and MG increased the formation of autophagosomes. The inhibition of lysosomal degradation using E64d and pepstatin A increased LC3-II levels in MG- or Vac-treated cells but not in Baf-treated cells (Fig. S3, *A*–*D*), suggesting that Baf suppressed lysosomal degradation. Lysosomal acidity, assayed using AcidiFluor ORANGE staining, was elevated in cells treated with MG and Vac but was decreased by Baf (Fig. 1, *D* and *E*). These results suggest the distinguishable effects of Baf and Vac on the inhibition of autolysosome formation.

Cells release EV with the different size and intracellular origin, and the proteins enriched within these vesicles differ between the small and large sizes (47). To quantify the amount of TDP-43 incorporated into the small- or large-sized EV, conditional media were subjected to sequential centrifugation at 20,000*g* (the P1 EV fraction) for the collection of larger vesicles, followed by 110,000*g* (the P2 EV fraction) for the collection of smaller vesicles. The expression levels of cellular proteins were quantified using the lysates of adhered cells, and the loading volumes of samples from the P1/P2 EV fractions were normalized by protein levels in the cell lysates. Immunoblotting results showed that the cleaved form of TDP-43 was detected by the anti-C-terminal TDP-43 antibody, but not by the anti-N-terminal TDP-43 antibody, suggesting that full-length and C-terminal TDP-43 proteins were both released by the Baf treatment (Fig. 1*F*). To differentiate the cleaved variants of TDP-43 present in the cell, P1, and P2 fractions, we subjected each fraction to immunoprecipitation using the polyclonal rabbit anti-C-terminal TDP-43 antibody. Samples were then immunoblotted with the monoclonal mouse anti-TDP-43 antibody. Immunoprecipitation analysis revealed the presence of multiple variants of cleaved TDP-43 proteins in the cell fraction as well as the P1 and P2 EV fractions (Fig. S4*A*). Four bands (a, b, c, and d) were detected in the cell fraction. Bands b and c were the predominant bands in the P1 EV fraction, whereas blurry band d was dominant in the P2 EV fraction (Fig. S4*B*). Densitometric data obtained from immunoblotting results on HeLa, SH-SY5Y neuroblastoma, and NSC-34 motor neuron–like cells showed that Baf, but not MG or Vac, significantly increased full-length and cleaved TDP-43 levels in the P1 and P2 EV fractions without affecting full-length TDP-43 expression levels in the cell fraction (Figs. 1, *G* and *H*, S5, and S6, *A*–*C*). The levels of the exosome marker TSG101 (tumor susceptibility gene 101 protein) in the P1 and P2 EV fractions were markedly increased by Baf but not by MG or Vac without any changes in the cellular expression levels (Figs. 1*I*, S5*B*, and S6, *A* and *D*). Baf, MG, and Vac increased the level of LC3-II, an autophagosome marker, in the cell fraction, whereas only Baf markedly increased LC3-II levels in the P1 and P2 EV fractions (Figs. 1*J*, S5*B*, and S6, *A* and *E*). These results suggest that the inhibition of autophagosome–lysosome fusion by Baf increased the secretion of full-length and C-terminal TDP-43 *via* vesicles associated with autophagosomes or exosomes.

### Formation and secretion of autophagy-associated vesicles are required for TDP-43 release

**Figure 2:**
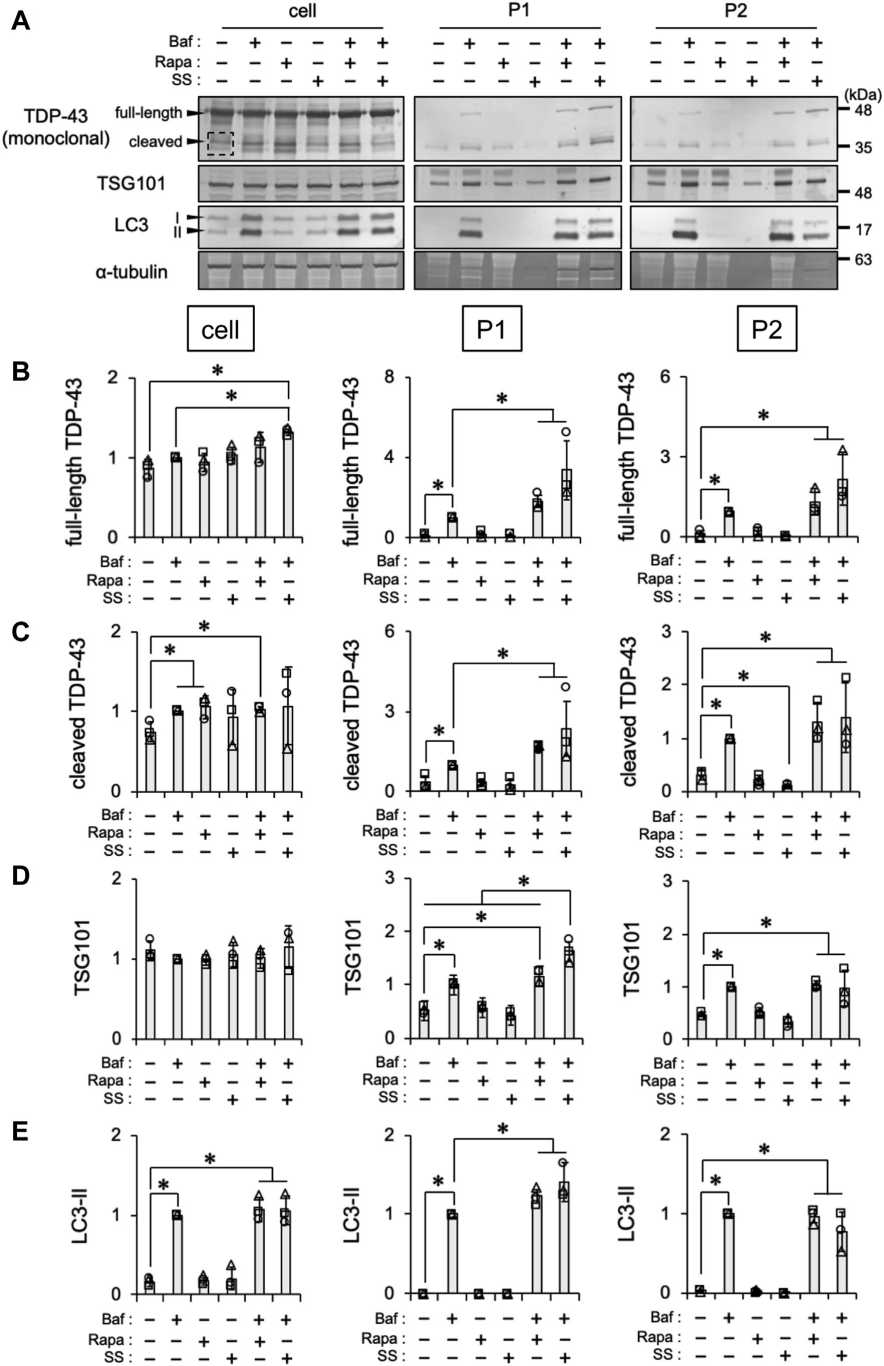
**Inhibition of autophagosome–lysosome fusion by bafilomycin A1 (Baf) is required for TDP-43 secretion *via* autophagy-associated vesicles.** HeLa cells were exposed to indicated treatment for 24 h. *A*, representative immunoblots (TDP-43 detected by the monoclonal antibody, TSG101, LC3, and α-tubulin) of each fraction are shown. The *dashed square* indicates bands of cleaved TDP-43 used for quantification. *B*–*E*, densitometric data on (*B*) full-length TDP-43, (*C*) cleaved TDP-43, (*D*) TSG101, and (*E*) LC3-II in the cell, P1, and P2 fractions were calculated from immunoblotting results. Relative signal intensities normalized against cells exposed to Baf are shown. In bar graphs, data are presented as means ± SD (N = 3). ∗ indicates *p* < 0.05 by the two-tailed unpaired *t* test. LC3, microtubule-associated proteins 1A/1B light chain 3B; Rapa, rapamaycin; SS, serum starvation; TDP-43, TAR DNA-binding protein 43 kDa; TSG101, tumor susceptibility gene 101 protein.

Baf increased the formation of autophagosomes under the inhibition of autophagosome–lysosome fusion. To investigate the relationship between EV-mediated TDP-43 secretion and autophagy, we examined the effects of autophagy inducers on TDP-43 secretion, phagophore formation, autophagic flux, and lysosomal degradation in the presence or the absence of Baf. HeLa cells were treated with Baf, Rapa, SS, Baf plus Rapa, or Baf plus SS for 24 h and then subjected to assays (Fig. 2*A*). Full-length TDP-43 levels in the cell fraction were higher in cells treated with Baf plus SS than in those treated with Baf only. On the other hand, cleaved TDP-43, TSG101, and LC3-II levels remained unchanged in the cell fractions of cells treated with Baf, Baf plus Rapa, and Baf plus SS (Fig. 2, *B*–*E*, cell). In the presence of Baf, the levels of full-length and cleaved TDP-43 and LC3-II were higher in the P1 EV fraction from cells treated with Rapa or SS compared with the untreated cells. The treatment with Baf plus SS increased TSG101 levels in the P1 EV fraction more than Baf alone or the Baf plus Rapa treatment (Fig. 2, *B*–*E*, P1). The levels of full-length and cleaved TDP-43, TSG101, and LC3-II in the P2 EV fraction were similar in cells treated with Baf, Baf plus Rapa, and Baf plus SS. SS significantly decreased cleaved TDP-43 levels in the P2 fraction (Fig. 2, *B*–*E*, P2). The autophagic flux assay using HeLa cells stably expressing GFP-LC3-RFP-LC3ΔG showed no change in the GFP/RFP ratio in response to the Rapa treatment and a decrease in response to SS, suggesting that SS, but not Rapa, facilitated autophagic flux (Fig. S1, *A* and *B*). WIPI2 immunostaining revealed that both Rapa and SS, similar to Baf, increased WIPI2 signal intensity. Moreover, the combination of Baf plus Rapa or Baf plus SS induced a larger increase in WIPI2 signal intensity than the single treatment with Baf, Rapa, or SS (Fig. S7, *A* and *B*). The inhibition of lysosomal degradation using E64d and pepstatin A increased LC3-II levels in cells treated with Rapa or SS, indicating that Rapa or SS did not suppress lysosomal degradation as effectively as Baf (Fig. S3, *A* and *B*). These results suggest a positive correlation between the levels of secreted TDP-43 and the secretion of autophagy-associated vesicles triggered by the inhibition of autophagosome–lysosome fusion.

### The Baf treatment facilitates the release of EV associated with autophagy

**Figure 3:**
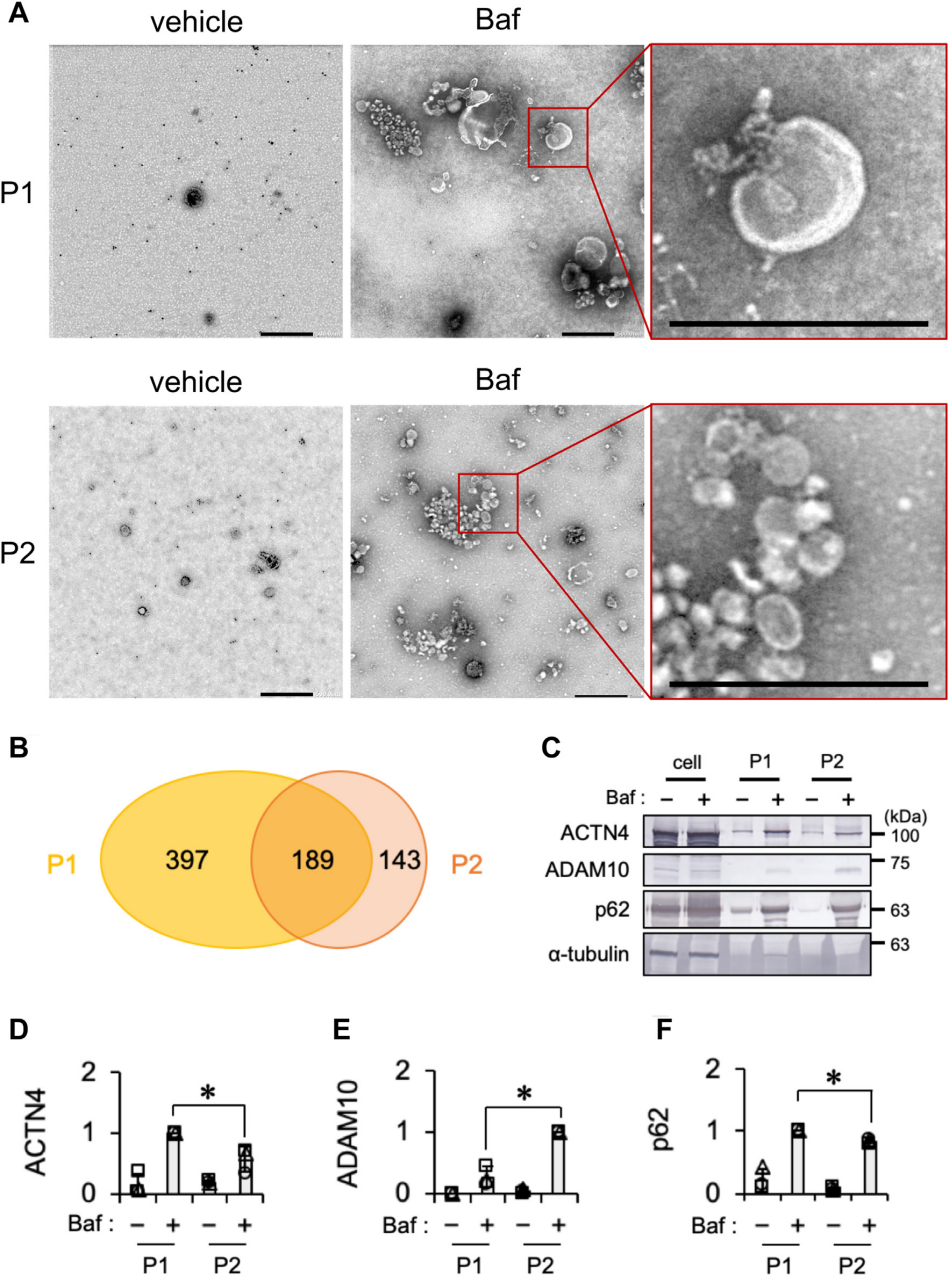
**Proteomic analyses suggest different intracellular origins of EV in the P1 and P2 fractions derived from HeLa cells exposed to bafilomycin A1 (Baf).** HeLa cells were exposed to vehicle (DMSO) or 100 nM Baf for 24 h. Cell, P1, and P2 fractions in each treatment were prepared. *A*, immuno-EM images of vesicles in the P1 and P2 fractions derived from cells exposed to 100 nM Baf using the rabbit anti-C-terminal TDP-43 antibody. The image surrounded by a *red brown square* was magnified to compare vesicles between the P1 and P2 fractions. Scale bar represents 500 nm. *B*, a Venn diagram showing the distribution of proteins in the P1 and P2 fractions identified by LC–MS/MS analyses. *C*, representative immunoblots (ACTN4, ADAM10, p62, and α-tubulin) of the cell, P1, and P2 fractions are shown. *D*–*F*, densitometric data on (*D*) ACTN4, (*E*) ADAM10, and (*F*) p62 in the P1 and P2 fractions were calculated from immunoblotting results. In bar graphs, data are presented as means ± SD (N = 3) normalized against the P1 or P2 fraction derived from cells exposed to Baf. ∗ indicates *p* < 0.05 by the two-tailed unpaired *t* test. DMSO, dimethyl sulfoxide; EV, extracellular vesicle; TDP-43, TAR DNA-binding protein 43 kDa.

We characterized the forms of vesicles in the P1 and P2 EV fractions derived from cells exposed to Baf. Electron microscopy revealed the presence of multiple vesicles in the P1 and P2 EV fractions obtained from cells treated with Baf but not in those obtained from untreated cells (Fig. 3*A*). Vesicles in the P1 EV fraction exhibited a wide range of sizes (approximately 50–1000 nm in diameter), whereas those in the P2 EV fraction were smaller (approximately 50–200 nm in diameter). Immunolabeling with the anti-C-terminal TDP-43 antibody confirmed the presence of both the full-length and cleaved forms of TDP-43. However, the TDP-43 fibril-like structures reported in a previous study (9) were not detected in the P1 or P2 EV fraction. The proteins present in vesicles were comprehensively identified by mass spectrometry. A Venn diagram shows the specific proteins detected solely in the P1 or P2 EV fraction as well as common proteins detected in both the P1 and P2 EV fractions by a proteomic analysis (Fig. 3*B* and Datasets S1–S3). A cellular component analysis using DAVID software (https://david.ncifcrf.gov/) (48) indicated different features among proteins detected in the P1 EV fraction only, the P2 EV fraction only, and in both the P1 and P2 EV fractions. Proteins involved in the terms “endoplasmic reticulum” and “sarcoplasmic reticulum” were detected in the P1 EV fraction only. On the other hand, proteins involved in the terms “membrane,” “Golgi apparatus,” “extracellular matrix,” “synapse,” “coated pit,” “membrane attack complex,” “LDL,” “chylomicron,” and “VLDL” were detected in the P2 EV fraction only (Table S1). Moreover, to confirm whether the difference in the size of vesicles between the P1 and P2 EV fractions reflected biochemical properties, as shown in a previous study (47), ACTN4 and ADAM10 levels were compared between the P1 and P2 EV fractions. ACTN4 is a protein that is enriched in large EV, whereas ADAM10 is enriched in small EV derived from endosomes or the plasma membrane (47). The Baf treatment increased ACTN4 and ADAM10 levels in both the P1 and P2 EV fractions. ACTN4 levels were significantly higher in the P1 EV fraction than in the P2 EV fraction, whereas ADAM10 levels were significantly higher in the P2 EV fraction than in the P1 EV fraction (Fig. 3, *C*–*E*), indicating that EV derived from cells exposed to Baf shared some of the properties with previously reported EV (47). The autophagic cargo adaptor p62 was enriched in both the P1 and P2 EV fractions. The level of p62 was slightly higher in the P1 EV fraction than in the P2 EV fraction (Fig. 3*F*), and the p62 band in the P2 EV fraction displayed a somewhat diffuse pattern (Fig. S8). These results suggest that the Baf treatment increased EV associated with autophagy in both the P1 and P2 EV fractions, originating from distinct sources.

### Loss of function of TDP-43 enhances LC3-II levels in EV induced by the Baf treatment

**Figure 4:**
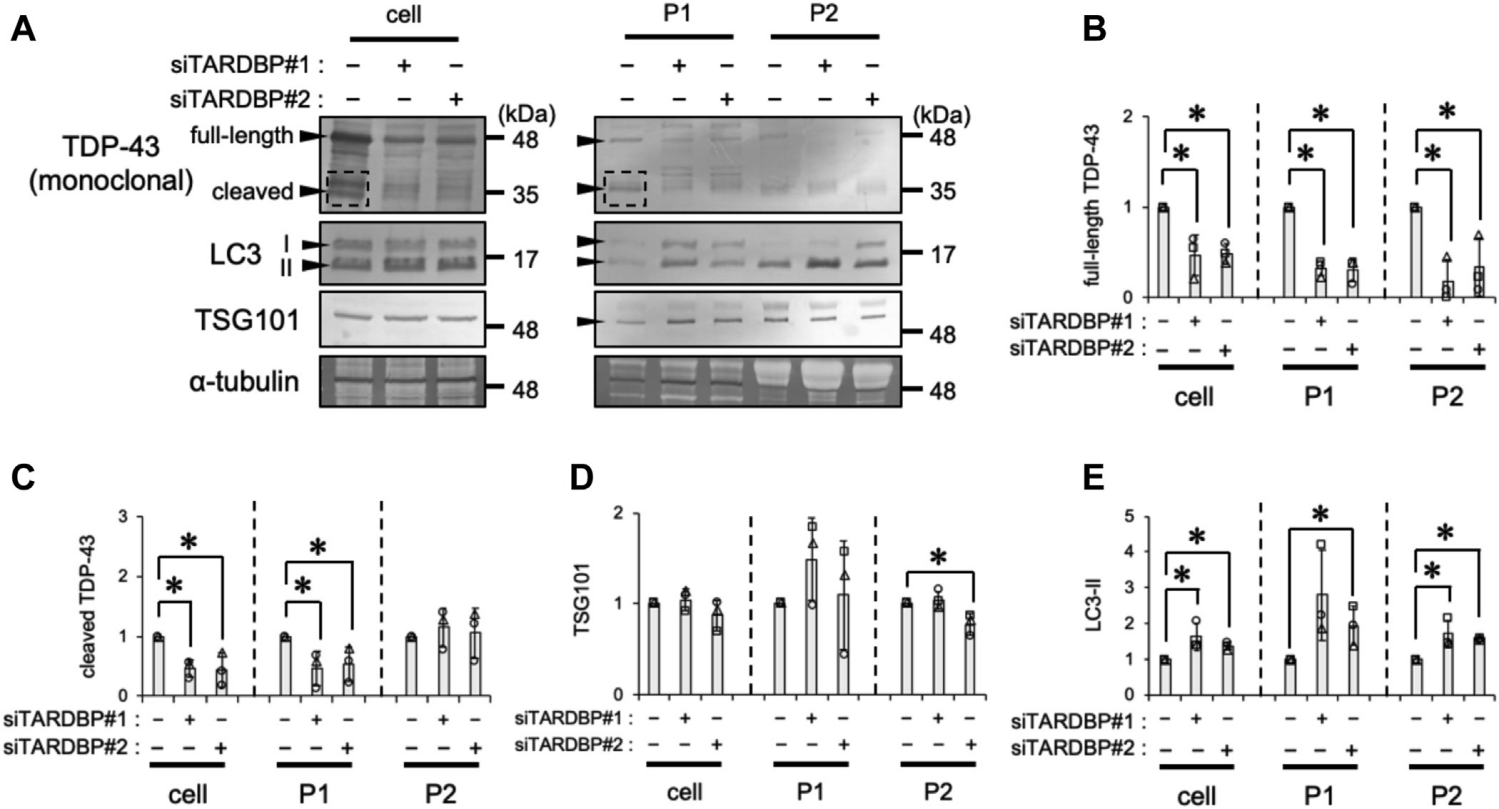
***TARDBP* knockdown enhances the extracellular release of LC3-II from bafilomycin A1 (Baf)–treated cells.** HeLa cells transfected with control or *TARDBP* siRNA for 48 h were further cultured in 100 nM Baf-containing medium for 24 h. The cell, P1, and P2 fractions were prepared as shown in the Experimental procedures section. *A*, representative immunoblots (TDP-43, TSG101, LC3, and α-tubulin) are shown. The *dashed square* indicates the bands of cleaved TDP-43 used for quantification. *B*–*E*, densitometric data on (*B*) full-length TDP-43, (*C*) cleaved TDP-43, (*D*) TSG101, and (*E*) LC3-II in each fraction were calculated from immunoblotting results. Relative signal intensities normalized against cells transfected with control siRNA are shown. In bar graphs, data are presented as means ± SD (N = 3). ∗ indicates *p* < 0.05 by the two-tailed unpaired *t* test. LC3, microtubule-associated proteins 1A/1B light chain 3B; TDP-43, TAR DNA-binding protein 43 kDa; TSG101, tumor susceptibility gene 101 protein.

The accumulation of the nuclear protein TDP-43 in the cytoplasm of an affected region in FTLD-TDP or ALS patients causes not only the toxic gain of function of TDP-43 in the cytoplasm but also the loss of function of TDP-43 in the nucleus (4). To mimic the loss of function of TDP-43, the TDP-43 gene (*TARDBP*) was silenced in HeLa cells using siRNA, and the Baf-induced secretion of autophagy-associated vesicles was then examined. As shown in Figure 4, *A* and *B*, full-length TDP-43 levels were significantly decreased in the cell, P1, and P2 fractions upon the knockdown of *TARDBP* (Fig. 4, *A* and *B*). The knockdown of *TARDBP* significantly decreased cleaved TDP-43 levels in the cell and P1 EV fractions but not in the P2 EV fraction (Fig. 4*C*). TSG101 levels in the cell and EV fractions were not affected by the loss of *TARDBP* (Fig. 4*D*). The levels of LC3-II were significantly increased not only in the cell fraction, as previously reported (49), but also in the P1 and P2 EV fractions (Fig. 4*E*). These results suggest that the loss of function of TDP-43 enhanced the cellular accumulation and the extracellular release of autophagy-associated vesicles induced by Baf.

### The C-terminal region of TDP-43 is required for Baf-induced TDP-43 secretion *via* EV

**Figure 5:**
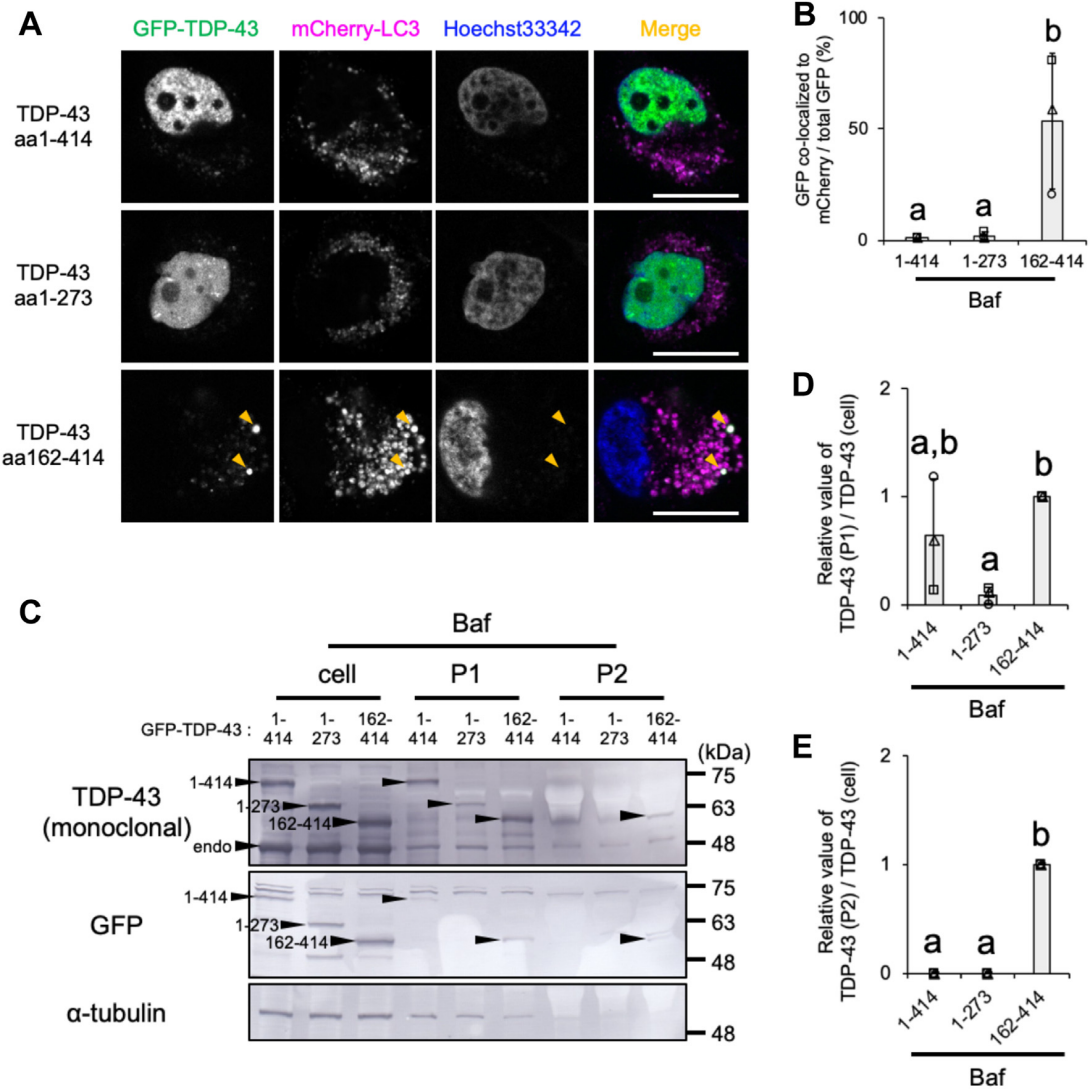
**The C-terminal region of TDP-43 is required for bafilomycin A1 (Baf)-induced TDP-43 secretion.***A* and *B*, HeLa cells stably expressing mCherry-LC3 transfected with GFP-tagged full-length (amino acids 1–414), the N-terminal fragment (amino acids 1–273), or C-terminal fragment (amino acids 162–414) of TDP-43 were exposed to 100 nM Baf for 24 h. *A*, representative confocal images of live cells in each treatment are shown. Nuclei were stained with Hoechst 33342. Scale bar represents 20 μm. *Arrowheads* indicate the colocalization of TDP-43 amino acids 162 to 414 and mCherry-LC3. *B*, densitometric data of fluorescence images were calculated from three independent experiments including at least 20 cells. The percentage of the GFP-TDP-43 signal colocalized with mCherry-LC3 to the total GFP-TDP-43 signal is shown. *C*–*E*, HeLa cells transfected with TDP-43 amino acids 1 to 414, TDP-43 amino acids 1 to 273, or TDP-43 amino acids 162 to 414 were exposed to 100 nM Baf for 24 h, and the cell, P1, and P2 fractions were prepared. *C*, representative immunoblots (TDP-43, GFP, and α-tubulin) of the cell, P1, and P2 fractions are shown. *D* and *E*, the ratio of GFP-TDP-43 levels in the (*D*) P1 and (*E*) P2 fractions to the cellular fraction calculated from the densitometric data of immunoblotting results are normalized against cells transfected with TDP-43 amino acids 162 to 414. In bar graphs, data are presented as means ± SD (N = 3). Values with a different superscript indicate *p* < 0.05 by Tukey’s post hoc test. LC3, microtubule-associated proteins 1A/1B light chain 3B; TDP-43, TAR DNA-binding protein 43 kDa.

We examined the structural element of TDP-43 that was required for incorporation into EV using already characterized GFP-tagged full-length TDP-43 (amino acids 1–414), GFP-tagged N-terminal TDP-43 (amino acids 1–273), or GFP-tagged C-terminal TDP-43 (amino acids 162–414) (50). HeLa cells stably expressing mCherry-LC3 were transiently transfected with GFP-TDP-43 proteins, and cells were treated with Baf (Fig. 5*A*). Confocal live-cell images showed the prominent nuclear localization of TDP-43 amino acids 1 to 414 and TDP-43 amino acids 1 to 273. In contrast, TDP-43 amino acids 162 to 414 colocalized with mCherry-LC3 (Fig. 5*B*). Sequential centrifugation was conducted to identify which regions of TDP-43 were required for the Baf-induced secretion of TDP-43 (Fig. 5*C*). Equivalent levels of the TDP-43 amino acids 1 to 414, TDP-43 amino acids 1 to 273, and TDP-43 amino acids 162 to 414 proteins were detected in the cell fraction. The P1 EV fraction had lower levels of TDP-43 amino acids 1 to 273 than those of TDP-43 amino acids 1 to 414 and TDP-43 amino acids 162 to 414 (Fig. 5C and *D*). Only TDP-43 amino acids 162 to 414 were detected in the P2 EV fraction (Fig. 5*E*). These results suggest that the C-terminal 162 to 414 region of TDP-43 was required for the Baf-induced secretion of TDP-43.

### The Baf treatment promotes the translocation of autophagosomes that carry TDP-43 to the plasma membrane

**Figure 6:**
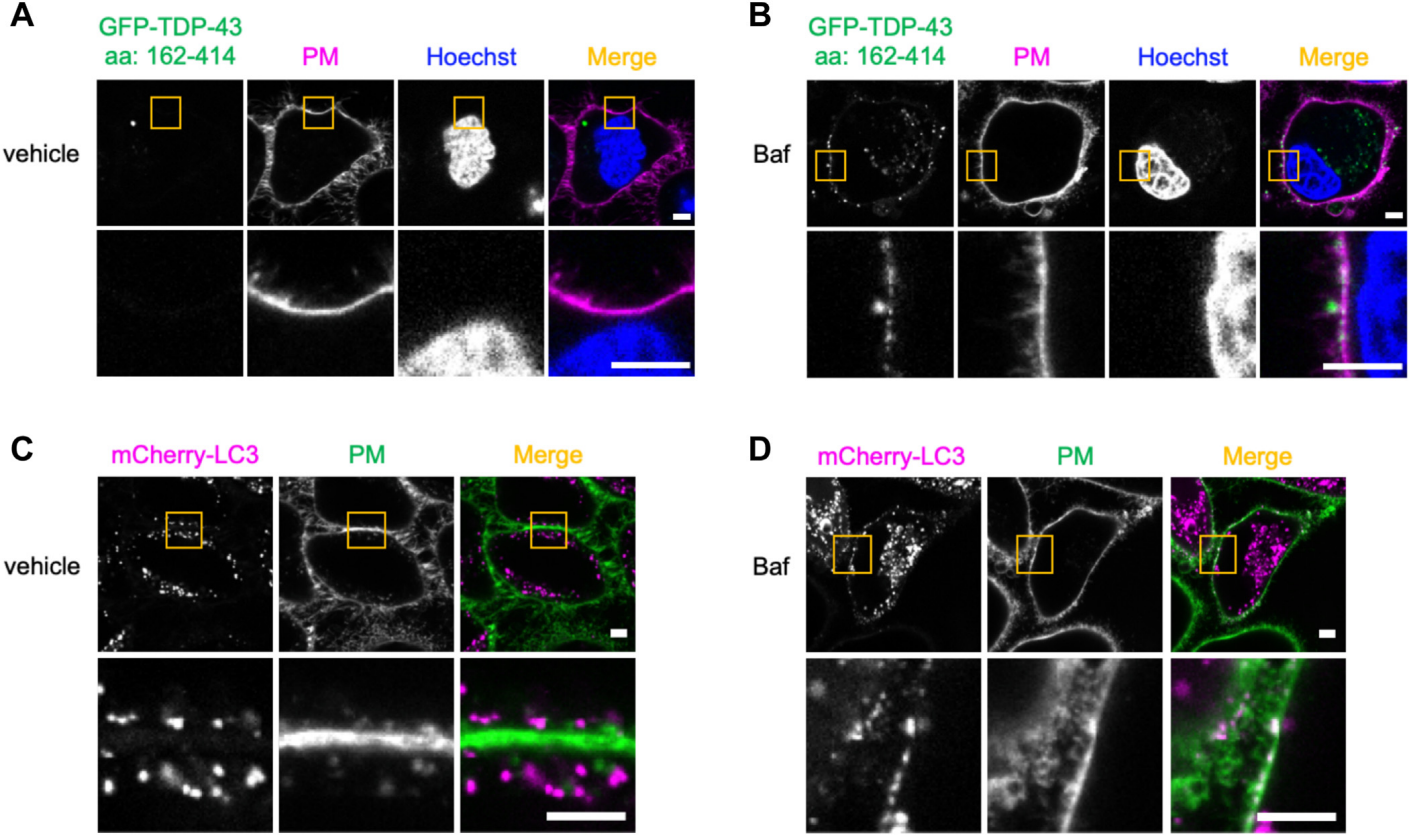
**The bafilomycin A1 (Baf) treatment induces the localization of GFP-tagged C-terminal TDP-43 (amino acids 162–414) and mCherry-LC3 to the plasma membrane.** HeLa cells transfected with the TDP-43 amino acids 162 to 414 construct or mCherry-LC3 construct were exposed to vehicle or 100 nM Baf for 24 h. *A* and *B*, representative confocal images of live cells transfected with the TDP-43 amino acids 162 to 414 construct (*green*) exposed to (*A*) vehicle or (*B*) 100 nM Baf are shown. The plasma membrane (PM) was stained with PlasMem Bright Red (*red*). Nuclei were stained with Hoechst 33342. *C* and *D*, representative confocal images of live cells transfected with the mCherry-LC3 construct (*red*) exposed to (*C*) vehicle or (*D*) 100 nM Baf are shown. The PM was stained with PlasMem Bright Green (*green*). The image surrounded by a *yellowsquare* was magnified. Scale bar represents 5 μm. LC3, microtubule-associated proteins 1A/1B light chain 3B; TDP-43, TAR DNA-binding protein 43 kDa.

The Baf-induced incorporation of TDP-43 in autophagosomes with the plasma membrane was monitored using confocal live-cell imaging. Without the Baf treatment, TDP-43 amino acids 162 to 414 and mCherry-LC3 were detected near the plasma membrane but clearly distal (Fig. 6, *A* and *C*). In contrast, when cells were treated with Baf, TDP-43 amino acids 162 to 414 inclusions larger than 0.2 μm in diameter (27) increased in the cytoplasm, and some TDP-43 amino acids 162 to 414 and mCherry-LC3 signals docked at the plasma membrane (Fig. 6, *B* and *D*). These results suggest that the Baf treatment facilitated the fusion of autophagosomes incorporating TDP-43 with the plasma membrane.

### ATG16L1 deficiency decreases the Baf-induced secretion of TDP-43 *via* EV

**Figure 7:**
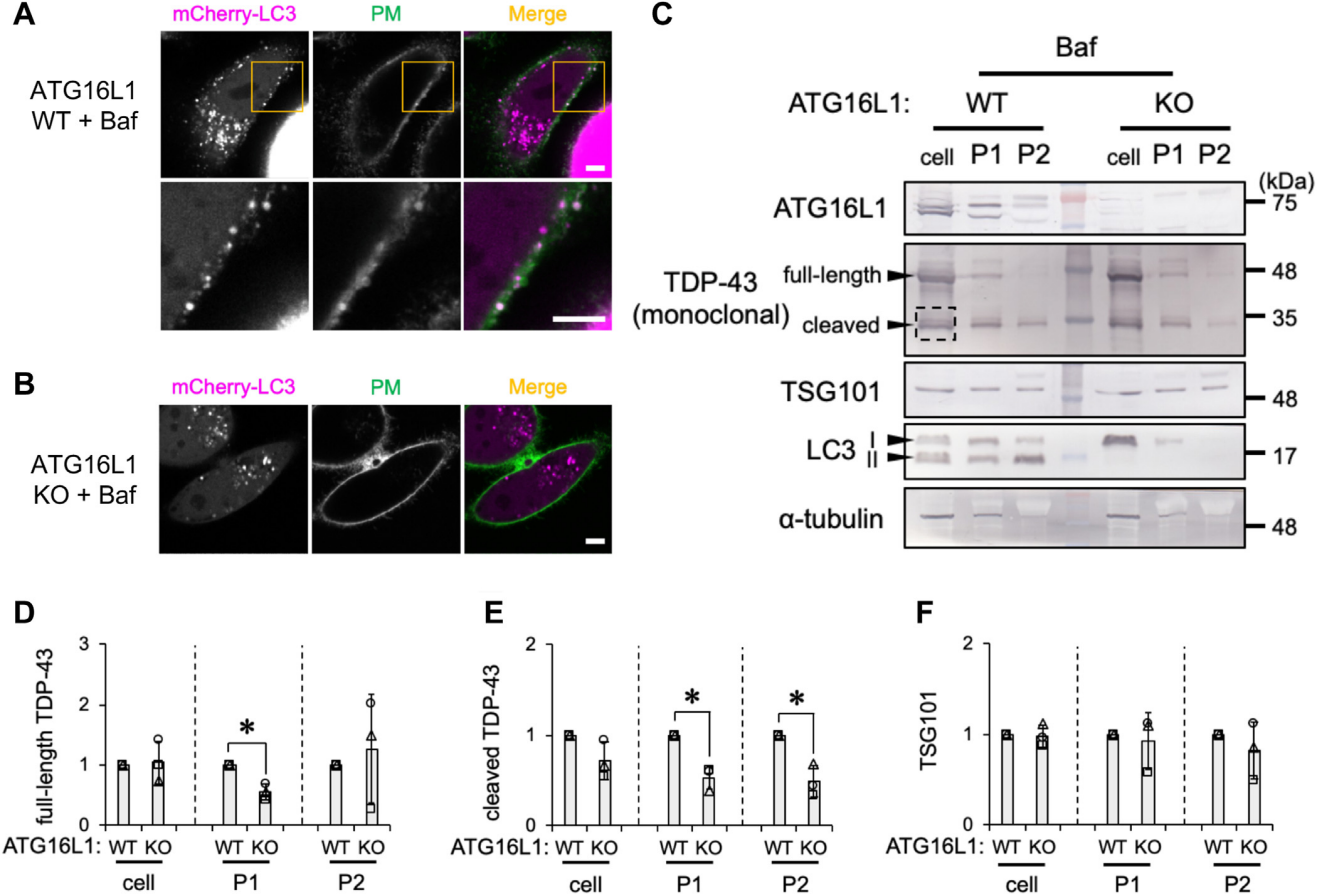
**ATG16L1 is required for bafilomycin A1 (Baf)-induced TDP-43 secretion.***A* and *B*, ATG16L1 WT or KO HeLa cells transfected with the mCherry-LC3 construct were exposed to 100 nM Baf for 24 h. Representative confocal live-cell images of ATG16L1 (*A*) WT or (*B*) KO cells transfected with the mCherry-LC3 (*red*) construct exposed to Baf. The plasma membrane (PM) was stained with PlasMem Bright Green (*green*). The image surrounded by a *yellowsquare* was magnified. Scale bar represents 5 μm. *C*–*F*, ATG16L1 WT or KO HeLa cells were exposed to 100 nM Baf for 24 h. The cell, P1, and P2 fractions were prepared as shown in the Experimental procedures section. *C*, representative immunoblots (ATG16L1, TDP-43, TSG101, LC3, and α-tubulin) of the cell, P1, and P2 fractions are shown. The *dashed square* indicates bands of cleaved TDP-43 used for quantification. *D*–*F*, densitometric data on (*D*) full-length TDP-43, (*E*) cleaved TDP-43, and (*F*) TSG101 in the cell, P1, and P2 fractions were calculated from immunoblotting results. Relative signal intensities normalized against ATG16L1 WT cells are shown. In bar graphs, data are presented as means ± SD (N = 3). ∗ indicates *p* < 0.05 by the two-tailed unpaired *t* test. LC3, microtubule-associated proteins 1A/1B light chain 3B; TDP-43, transactive response DNA-binding protein 43 kDa; TSG101, tumor susceptibility gene 101 protein.

The loss of cellular ATG16L1 expression has been shown to suppress the formation of autophagosomes (51). Therefore, we investigated whether autophagy mediated the Baf-induced secretion of TDP-43 using ATG16L1 KO autophagy-null HeLa cells (51). Under the Baf treatment, the mCherry-LC3 signal was detected within the plasma membrane of control HeLa cells but was distributed in the cytoplasm of ATG16L1-KO cells (Fig. 7, *A* and *B*). A deficiency of autophagosomes was confirmed by the loss of LC3-II production in ATG16L1 KO cells (Fig. 7*C*). Densitometric data revealed that ATG16L1 KO attenuated the incorporation of full-length TDP-43 in P1 vesicles and cleaved TDP-43 in P1 and P2 vesicles (Fig. 7, *D* and *E*). On the other hand, ATG16L1 KO did not affect TSG101 levels in the cell or EV fraction (Fig. 7*F*). These results suggest that the formation of autophagosomes was necessary for the Baf-induced secretion of both the full-length and cleaved TDP-43 proteins.

### Blocking autophagosome–lysosome fusion does not induce TDP-43 secretion *via* EV

**Figure 8:**
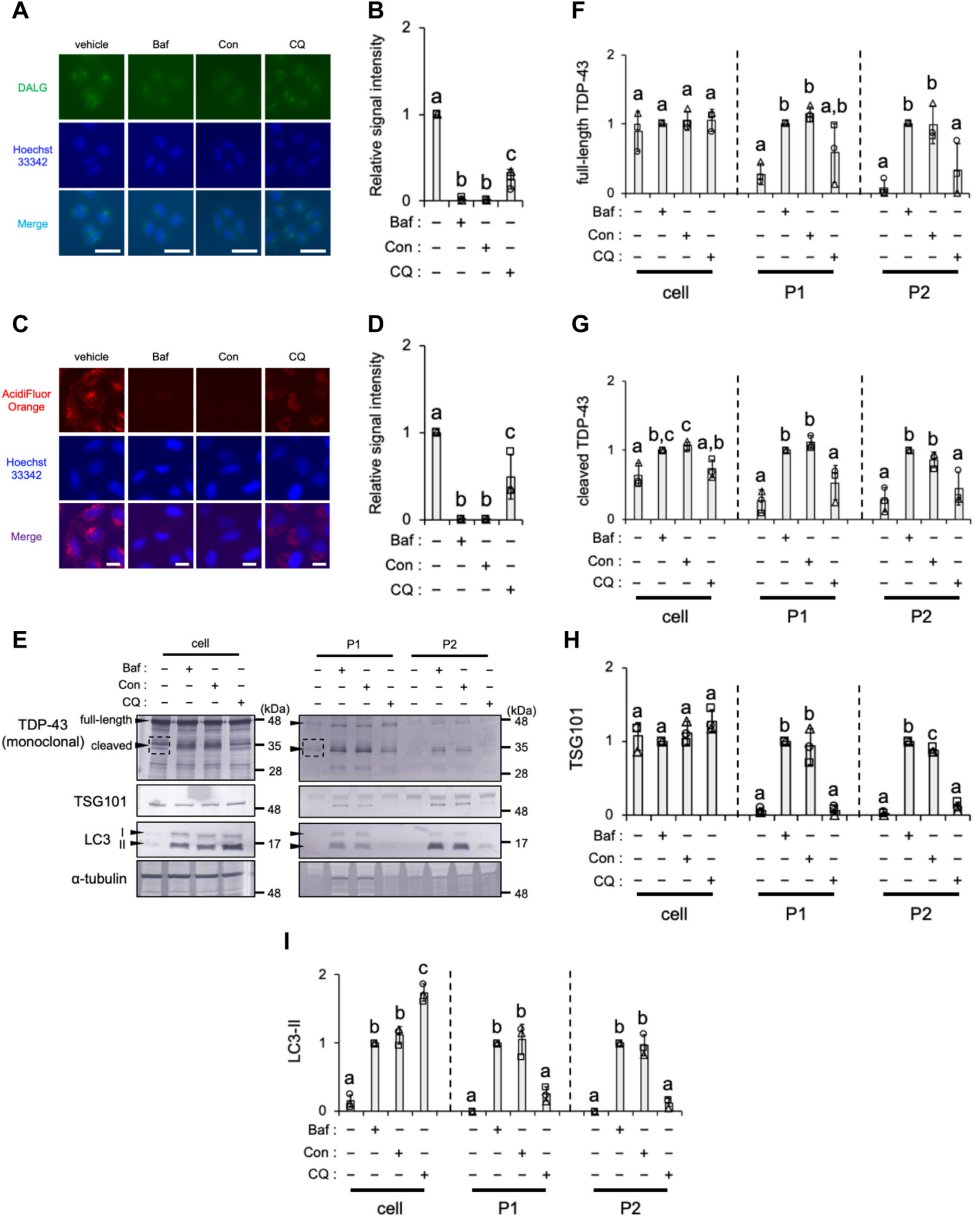
**Chloroquine, an inhibitor of autolysosome formation and lysosomal acidification, does not increase the secretion of TDP-43 or LC3-II.***A* and *B*, HeLa cells stained with DALGreen were exposed to vehicle (DMSO), 100 nM Baf, 100 nM Con, or 100 μM CQ for 2 h. *A*, representative images of live cells stained with DALGreen (*green*) in each treatment are shown. Nuclei were stained with Hoechst 33342 (*blue*). Scale bar represents 50 μm. *B*, relative signal intensities normalized against cells exposed to vehicle were calculated from three independent experiments including at least 74 cells. *C* and *D*, HeLa cells exposed to vehicle (DMSO), 100 nM Baf, 100 nM Con, or 100 μM CQ for 24 h were stained with AcidiFluor ORANGE. *C*, representative images of live cells stained with AcidiFluor ORANGE (*red*) in each treatment. Nuclei were stained with Hoechst 33342 (*blue*). Scale bar represents 20 μm. *D*, relative signal intensities normalized against cells exposed to vehicle were calculated from three independent experiments including at least 332 cells. *E*–*I*, HeLa cells were exposed to vehicle (DMSO), 100 nM Baf, 100 nM Con, or 100 μM CQ for 24 h. The cell, P1, and P2 fractions were prepared as shown in the Experimental procedures section. *E*, representative immunoblots (TDP-43, TSG101, LC3, and α-tubulin) of the cell, P1, and P2 fractions are shown. The *dashed square* indicates the bands of cleaved TDP-43 used for quantification. *F*–*I*, densitometric data on (*F*) full-length TDP-43, (*G*) cleaved TDP-43, (*H*) TSG101, and (*I*) LC3-II were calculated from immunoblotting results. Relative signal intensities normalized against cells exposed to the vehicle are shown. In bar graphs, data are presented as means ± SD (N = 3). Values with a different superscript indicate *p* < 0.05 by Tukey’s post hoc test. Baf, bafilomycin A1; Con, concanamycin A; CQ, chloroquine; DMSO, dimethyl sulfoxide; LC3-II, microtubule-associated proteins 1A/1B light chain 3B; TDP-43, transactive response DNA-binding protein 43 kDa; TSG101, tumor susceptibility gene 101 protein.

We investigated the mechanisms underlying the secretion of TDP-43 using Con, the inhibitor of v-ATPase and autophagosome–lysosome fusion, or CQ, the inhibitor of autophagosome–lysosome fusion. The autophagic flux was suppressed in HeLa cells stably expressing GFP-LC3-RFP-LC3ΔG when treated with either 100 nM Con or 100 μM CQ as indicated by an increased GFP/RFP ratio (Fig. S1, *A*–*D*). The formation of autolysosomes indicated by DALGreen (DALG) was also significantly suppressed by 100 nM Con, 100 μM CQ, or 100 nM Baf (Fig. 8, *A* and *B*). These results suggest that both Con and CQ suppressed autophagosome–lysosome fusion, similar to Baf. On the other hand, the Con and CQ treatments increased WIPI2 signal intensity, which suggested that they promoted autophagosome formation, similar to Baf (Fig. S2, *A* and *B*). In parallel, lysosomal acidity was suppressed by the treatment with 100 nM Con, 100 μM CQ, or 100 nM Baf as assessed by AcidiFluor ORANGE (Fig. 8, *C* and *D*). The treatment with 100 nM Con increased the levels of both full-length and cleaved TDP-43 proteins in the P1 and P2 EV fractions, as did treatment with 100 nM Baf, but 10 or 100 μM CQ failed to induce the secretion of TDP-43 (Figs. 8, *E*–*G*, and S9). Unlike 100 nM Baf or Con, the CQ treatment failed to increase TSG101 and LC3-II levels in the P1 and P2 EV fractions but increased LC3-II levels in the cell fraction (Figs. 8, *E*, *H*, and *I*, S4, and S9). At a higher dose of 1 mM CQ, cells detached from culture dishes. Because of its inability to enhance exocytotic activity, CQ did not appear to effectively facilitate the secretion of TDP-43.

The roles of autophagosome–lysosome fusion in the secretion of TDP-43 were further examined in HeLa cells by the siRNA knockdown of the syntaxin 17 gene (*STX17*), which encodes a SNARE (soluble NSF attachment protein receptor) protein that regulates autophagosome–lysosome fusion (52). The knockdown of *STX17* reduced autolysosome formation indicated by the signals of DALG (Fig. S10, *A* and *B*) and lysosomal acidification indicated by the signal of AcidiFluor ORANGE (Fig. S10, *C* and *D*), consistent with previous reports (53, 54). However, the knockdown of *STX17* failed to increase full-length or cleaved TDP-43 levels in the P1 and P2 EV fractions, whereas full-length TDP-43 levels in the cell fraction were slightly increased (Fig. S10, *E*–*G*). Similarly, the knockdown of *STX17* significantly increased LC3-II levels in the cell fraction but not in the P1 or P2 EV fraction (Fig. S10, *E* and *H*). These results suggest that the suppression of autophagosome–lysosome fusion did not contribute to increases in the secretion of TDP-43.

### A PGRN insufficiency increases cleaved TDP-43 levels in EV fractions in an autophagy-dependent manner

**Figure 9:**
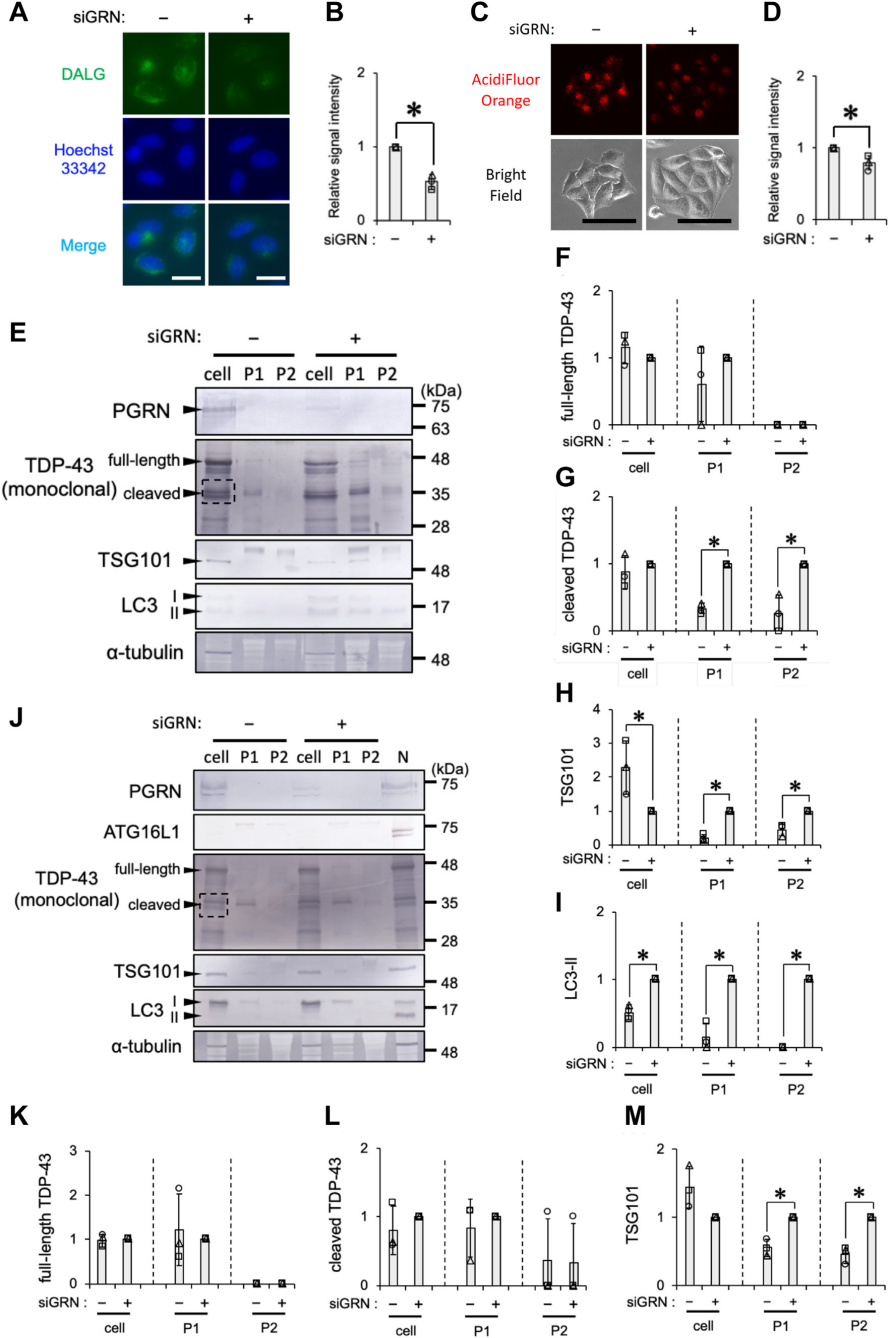
**The knockdown of *GRN* increases the secretion of TDP-43 and LC3-II in an autophagy-dependent manner.***A* and *B*, HeLa cells transfected with control or *GRN* siRNA for 48 h were stained with DALGreen and incubated for 2 h. *A*, representative live-cell images stained with DALGreen (*green*) are shown. Nuclei were stained with Hoechst 33342 (*blue*). Scale bar represents 50 μm. *B*, relative signal intensities normalized against cells transfected with control siRNA were calculated from three independent experiments including at least 78 cells. *C* and *D*, HeLa cells transfected with control or *GRN* siRNA for 48 h were stained with AcidiFluor ORANGE, and the acidity of lysosomes was compared. *C*, representative live-cell images stained with AcidiFluor ORANGE and photographed at a bright field are shown. Scale bar represents 100 μm. *D*, relative signal intensities normalized against cells transfected with control siRNA were calculated from three independent experiments including at least 627 cells. *E*–*I*, HeLa cells transfected with control or *GRN* siRNA were incubated for 72 h. The cell, P1, and P2 fractions were prepared as shown in the Experimental procedures section. *E*, representative immunoblots (PGRN, TDP-43, TSG101, LC3, and α-tubulin) of the cell, P1, and P2 fractions are shown. The *dashed square* indicates the bands of cleaved TDP-43 used for quantification. *F*–*I*, densitometric data on (*F*) full-length TDP-43, (*G*) cleaved TDP-43, (*H*) TSG101, and (*I*) LC3-II were calculated from immunoblotting results. Relative signal intensities normalized against cells transfected with *GRN* siRNA are shown. *J*–*M*, ATG16L1 KO HeLa cells transfected with control or *GRN* siRNA were incubated for 72 h. The cell, P1, and P2 fractions were prepared as shown in the Experimental procedures section. N is a sample from normal HeLa cells. *J,* representative immunoblots (PGRN, ATG16L1, TDP-43, TSG101, LC3, and α-tubulin) of the cell, P1, and P2 fractions are shown. The *dashed square* indicates the bands of cleaved TDP-43 used for quantification. *K*–*M*, densitometric data on (*K*) full-length TDP-43, (*L*) cleaved TDP-43, and (*M*) TSG101 were calculated from immunoblotting results. Relative signal intensities normalized against cells transfected with *GRN* siRNA are shown. In bar graphs, data are presented as means ± SD (N = 3). ∗ indicates *p* < 0.05 by the two-tailed unpaired *t* test or Mann–Whitney *U* test. LC3, microtubule-associated proteins 1A/1B light chain 3B; PGRN, progranulin; TDP-43, transactive response DNA-binding protein 43 kDa; TSG101, tumor susceptibility gene 101 protein.

PGRN regulates the autophagy-lysosomal pathway, and its insufficiency causes FTLD-TDP (37, 40, 55). We investigated whether a PGRN insufficiency facilitated TDP-43 secretion using the siRNA knockdown of the PGRN gene (*GRN* or *Grn*) in HeLa cells or NSC-34 motor neuron–like cells. The knockdown of *GRN* significantly decreased DALG (Fig. 9, *A* and *B*) and AcidiFluor ORANGE (Fig. 9, *C* and *D*) signal intensities, indicating the suppression of autolysosome formation and lysosomal acidification. The knockdown of *GRN* increased the levels of the cleaved TDP-43 protein in the P1 and P2 EV fractions, whereas full-length TDP-43 levels remained unchanged in all three fractions of HeLa cells (Fig. 9, *E*–*G*). Furthermore, the knockdown of *GRN* increased TSG101 levels in the P1 and P2 EV fractions but decreased them in the cell fraction (Fig. 9, *E* and *H*). In addition, the knockdown of *GRN* increased LC3-II levels in the cell and EV fractions (Fig. 9, *E* and *I*). Importantly, in NSC-34 cells, which exhibit a lower abundance of autophagosome-like particles (Fig. S6*F*), the knockdown of *Grn* increased cleaved TDP-43 levels in the P2 EV fraction, mimicking the effects observed in HeLa cells (Fig. S11). Therefore, the loss of PGRN expression appeared to promote the release of TDP-43-containing EV from both HeLa and NSC-34 cells. In NSC-34 cells, the levels of TSG101 and LC3-II were not changed by the knockdown of Grn, suggesting the different roles of PGRN in secretion, compared with HeLa cells (Fig. S11, *E* and *F*). To examine the role of autophagy in the *GRN* knockdown–induced secretion of TDP-43, we conducted assays using ATG16L1 KO HeLa cells (Fig. 9*J*). Without the expression of ATG16L1, the knockdown of *GRN* no longer induced the secretion of the full-length and cleaved TDP-43 proteins into the P1 and P2 EV fractions. On the other hand, it significantly increased TSG101 levels in the EV fraction, even in ATG16L1-deficient cells (Fig. 9, *J*–*M*).

**Figure 10:**
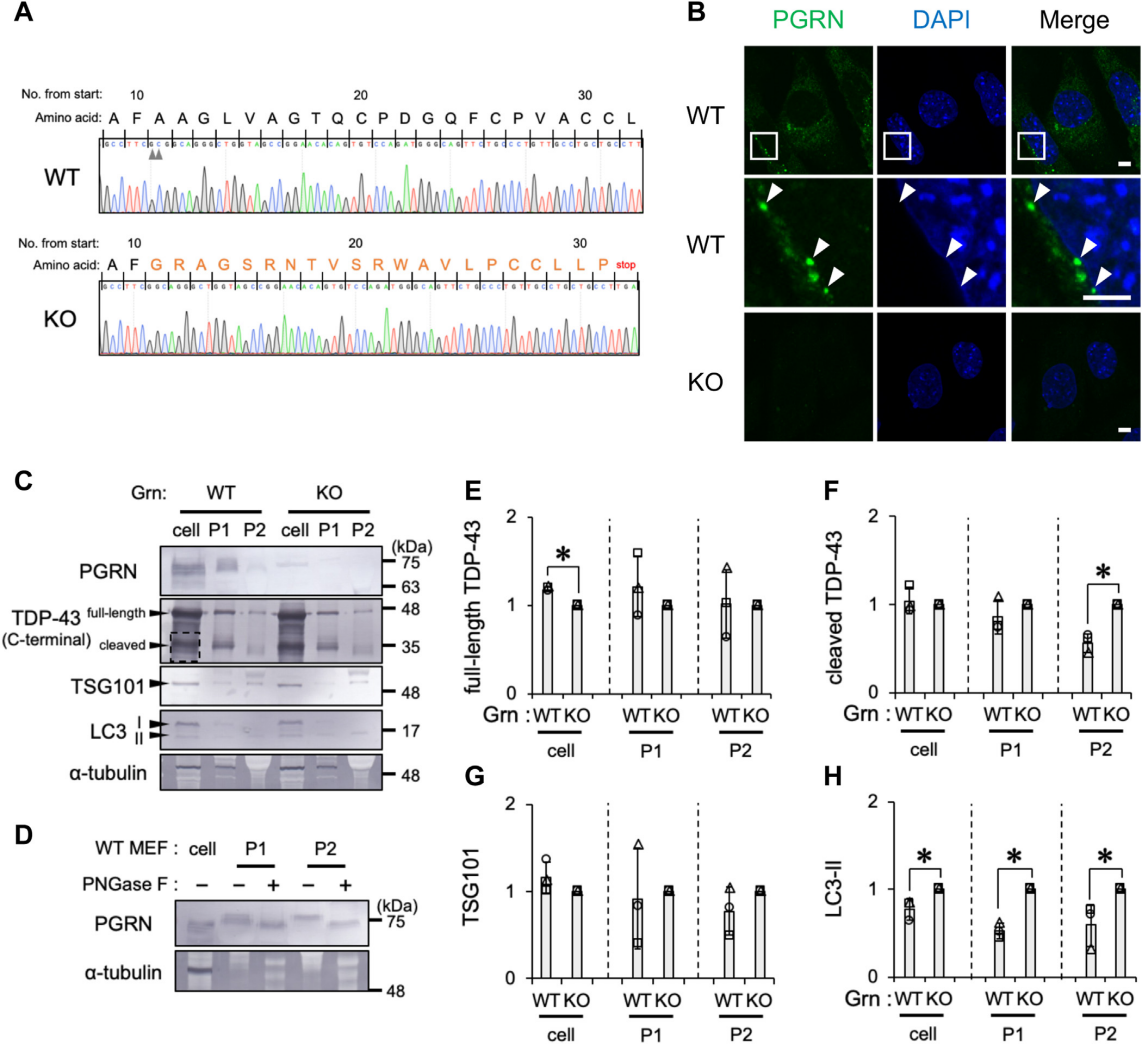
**The KO of *Grn* increases the secretion of TDP-43 and LC3-II from MEFs.***A*, the base sequences of the genome-edited regions of PGRN in WT and KO mice were elucidated by Sanger sequencing. *Arrowheads* in the WT alignment show the two bases deleted by CRISPR–Cas9 genome editing. *B*, representative confocal images of PGRN (*green*) immunostaining of MEFs derived from PGRN WT and KO mice. Nuclei were stained with DAPI (*blue*). *Arrowheads* indicate vesicle-like structures. The image surrounded by a *white square* in WT MEFs was magnified. Scale bar represents 5 μm. *C*–*H*, PGRN WT and KO MEFs were cultured for 72 h. The cell, P1, and P2 fractions were prepared as shown in the Experimental procedures section. *C*, representative immunoblots (PGRN, TDP-43, TSG101, LC3, and α-tubulin) of the cell, P1, and P2 fractions are shown. The *dashed square* indicates bands of cleaved TDP-43 used for quantification. *D*, the P1 and P2 fractions treated with or without PNGase F were immunoblotted with PGRN and α-tubulin antibodies. Representative immunoblots of the cell, P1, and P2 fractions are shown (N = 3). *E*–*H*, densitometric data on (*E*) full-length TDP-43, (*F*) cleaved TDP-43, (*G*) TSG101, and (*H*) LC3-II were calculated from immunoblotting results. Relative signal intensities normalized against PGRN KO MEFs are shown. In bar graphs, data are presented as means ± SD (N = 3). ∗ indicates *p* < 0.05 by the two-tailed unpaired *t* test. DAPI, 4′,6-diamidino-2-phenylindole; LC3-II, microtubule-associated proteins 1A/1B light chain 3B; MEF, mouse embryonic fibroblast; PGRN, progranulin; TDP-43, transactive response DNA-binding protein 43 kDa; TSG101, tumor susceptibility gene 101 protein.

To further validate the role of PGRN in the secretion of cleaved TDP-43 and LC3-II, we used MEFs derived from PGRN WT or KO mice (Fig. 10*A*). In WT MEFs, PGRN localized at vesicle-like structures (Fig. 10*B*, *arrowheads*) and was detected in the cell and EV fractions (Fig. 10*C*). Notably, the molecular weight of PGRN was higher in the P1 and P2 EV fractions than in the cell fraction (Fig. 10*C*). The deglycosylation of the P1 and P2 EV fractions using PNGase F resulted in a downward shift of the PGRN band to a similar position to that of the cell fraction (Fig. 10*D*). Therefore, PGRN included in EV was highly glycosylated. The loss of PGRN expression significantly reduced full-length TDP-43 levels in the cell fraction and increased cleaved TDP-43 levels in the P2 EV fraction (Fig. 10, *C*, *E*, and *F*). The level of LC3-II, but not TSG101, was elevated in all three fractions of PGRN-null cells (Fig. 10, *C*, *G*, and *H*). These results suggest that the PGRN insufficiency increased the secretion of TDP-43 in an autophagy-dependent manner.

### TDP-43 secretion *via* EV correlates with the nuclear localization of transcription factor EB

**Figure 11:**
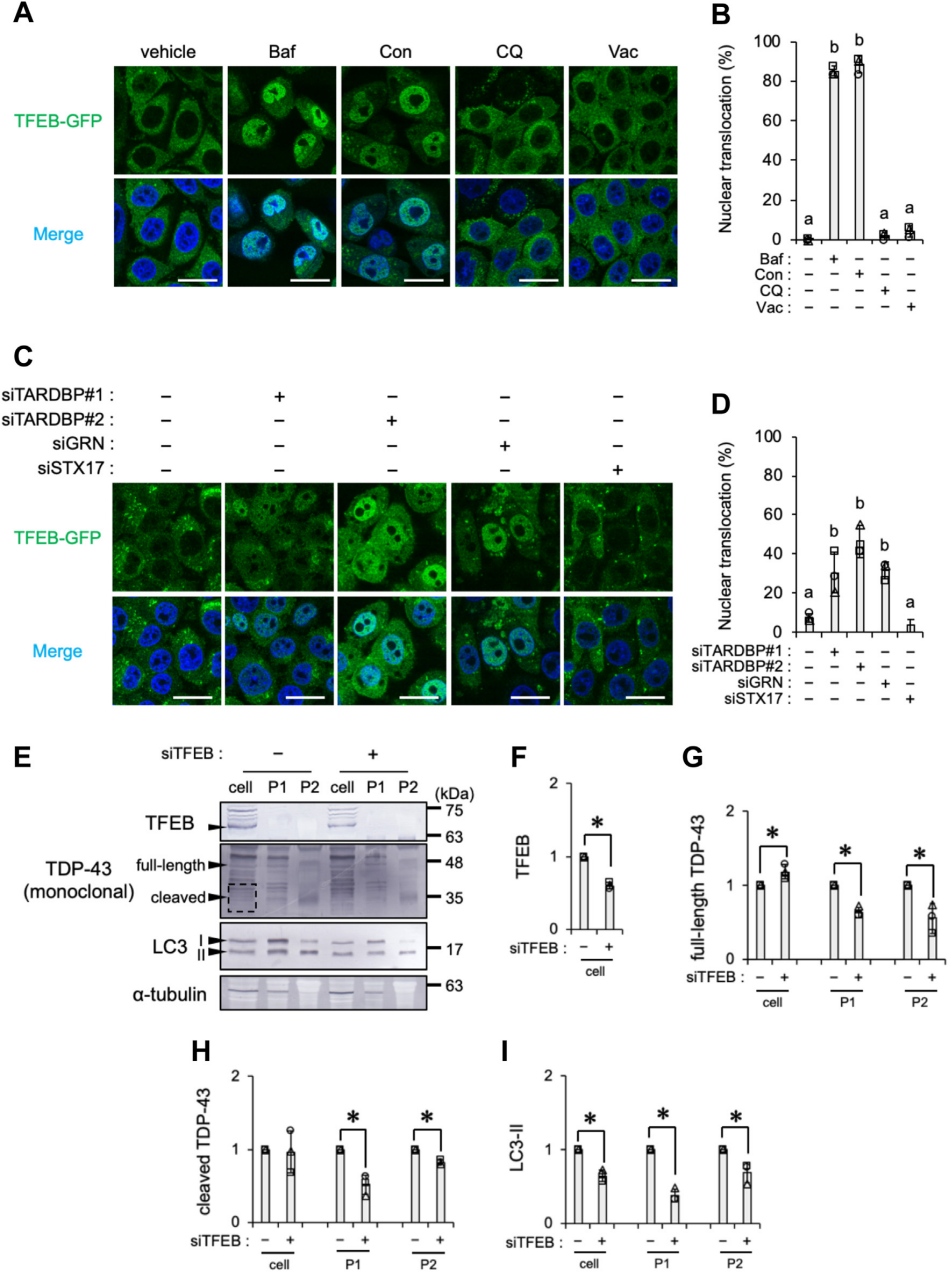
**Treatments leading to the secretion of TDP-43 promote TFEB-GFP nuclear localization in SH-SY5Y cells.***A* and *B*, SH-SY5Y cells stably expressing TFEB-GFP were exposed to DMSO, 100 nM Baf, 100 nM Con, 100 μM CQ, or 100 nM Vac for 24 h. *A*, representative confocal images are shown. Nuclei were stained with DAPI (*blue*). Scale bar represents 20 μm. *B*, the percentage of cells in which TFEB-GFP translocated in the nucleus calculated from three independent experiments including at least 102 cells is shown. *C* and *D*, SH-SY5Y cells stably expressing TFEB-GFP were transfected with control, *TARDBP* #1, *TARDBP* #2, *GRN*, or *STX17* siRNA for 48 h. *C*, representative confocal images are shown. Nuclei were stained with DAPI (*blue*). Scale bar represents 20 μm. *D*, the percentage of cells in which TFEB-GFP translocated in the nucleus calculated from three independent experiments including at least 120 cells is shown. *E*–*I*, SH-SY5Y cells transfected with control or *TFEB* siRNA were incubated for 72 h. The cell, P1, and P2 fractions were prepared as shown in the Experimental procedures section. *E*, representative immunoblots (TFEB, TDP-43, LC3, and α-tubulin) of the cell, P1, and P2 fractions are shown. The *dashed square* indicates the bands of cleaved TDP-43 used for quantification. *F*–*I*, densitometric data on (*F*) TFEB, (*G*) full-length TDP-43, (*H*) cleaved TDP-43, and (*I*) LC3-II were calculated from immunoblotting results. Relative signal intensities normalized against cells transfected with control siRNA are shown. In bar graphs, data are presented as means ± SD (N = 3). Values with a different superscript indicate *p* < 0.05 by Tukey’s post hoc test. ∗ indicates *p* < 0.05 by the two-tailed unpaired *t* test. Baf, bafilomycin A1; Con, concanamycin A; CQ, chloroquine; DAPI, 4′,6-diamidino-2-phenylindole; DMSO, dimethyl sulfoxide; TDP-43, transactive response DNA-binding protein 43 kDa; TFEB, transcription factor EB; Vac, vacuolin-1.

The present results suggest that the inhibition of autolysosome formation followed by lysosomal acidification plays a role in the secretion of TDP-43. On the other hand, the treatment with CQ and knockdown of STX17, each of which also suppressed autolysosome formation, were insufficient to promote TDP-43 secretion, suggesting the involvement of other factors in TDP-43 secretion. We previously reported that a treatment with Baf increased the nuclear localization of transcription factor EB (TFEB), a master regulator of lysosomal biogenesis and autophagy (29, 56). Furthermore, PGRN deficiency enhanced the nuclear localization of TFEB/TFE3 upon the accumulation of lysosomal proteins (35, 57). To investigate the potential relationship between the nuclear localization of TFEB and the secretion of TDP-43, SH-SY5Y cells stably expressing TFEB-GFP, a previously characterized cell line, were employed (29, 58). Under conditions inducing TDP-43 secretion, such as the treatment with 100 nM Baf or Con (Fig. 11, *A* and *B*) or the knockdown of *TARDBP* or *GRN* (Fig. 11, *C* and *D*), TFEB-GFP translocated to the nucleus. In contrast, treatments that did not promote TDP-43 secretion, such as 100 μM CQ, 100 nM Vac, or the knockdown of *STX17*, did not lead to the nuclear localization of TFEB-GFP (Fig. 11, *A*–*D*). Therefore, TDP-43 secretion appeared to correlate with the nuclear localization of TFEB. Conversely, we investigated whether the knockdown of *TFEB* interfered with the Baf-induced secretion of TDP-43. The knockdown of *TFEB* using siRNA decreased the levels of both full-length and cleaved TDP-43 in the P1 and P2 EV fractions, whereas it increased the levels of full-length TDP-43 in the cell fraction. Consistently, the knockdown of *TFEB* decreased LC3-II levels in the cell and EV fractions (Fig. 11, *E*–*I*). These results suggest that an increase in the nuclear translocation of TFEB is a key event for the secretion of TDP-43 *via* EV release.

## Discussion

In the brains of ALS and FTLD-TDP patients, the pathological accumulation of TDP-43 is initially deposited in specific areas and then spreads widely throughout the brain with disease progression (11). The C-terminal domain of TDP-43 serves as the core region of a fibrous structure, leading to the self-templating aggregation of TDP-43 (8) in the brains of ALS and FTLD-TDP patients (9). Moreover, distinct C-terminal fragments of TDP-43 with the specific structure for ALS or FTLD-TDP patients have been detected in multiple brain regions and the spinal cords of ALS and FTLD-TDP patients (5, 9, 59, 60), suggesting that TDP-43 with the same pathological structure is propagated to different brain regions in a prion-like manner. Thus, to prevent this prion-like spreading of TDP-43 (11), it is imperative to clear the cell-to-cell spreading mechanism of TDP-43.

We herein investigated the mechanisms underlying the secretion of TDP-43 and revealed that the secretion of TDP-43 *via* EV was regulated by autophagy. Inhibitors of v-ATPase, such as Baf and Con, suppressed autophagy but facilitated the secretion of endogenous TDP-43. On the other hand, MG, an inhibitor of proteasome degradation, facilitated autophagy and did not affect the secretion of endogenous TDP-43. In contrast, Iguchi *et al*. (26) reported that treatments with Baf and MG both increased ectopically expressed TDP-43 levels in the exosome fraction. Collectively, the present results and previous findings showed that EV release was promoted by Baf or Con but not by MG (28). This discrepancy may be due to overexpression of TDP-43 stimulating other secretory pathways (61), or it could be a result of increased TDP-43 levels because of overexpression leading to additional secretion. Under Baf-treated condition, mCherry-tagged LC3-II-positive puncta and GFP-tagged C-terminal fragment of TDP-43 often colocalized, and they also colocalized with the plasma membrane. When the formation of autophagosomes was inhibited because of the loss of ATG16L1 expression, the secretion of TDP-43 was suppressed without affecting exosome release. Therefore, it appears that Baf treatment induces the secretion of TDP-43 through autophagy-regulated vesicles.

Both Baf and Con inhibited lysosomal acidification and autolysosome formation and also stimulated the secretion of TDP-43. Previous studies reported the inhibition of lysosomal acidification by Baf and Con through the inhibition of v-ATPase (62, 63). Baf was also shown to suppress the formation of autolysosomes through the inhibition of a Ca^2+^-transporting ATPase (64). In contrast, Vac, MG, CQ, and the knockdown of *STX17*, all of which increased the cellular accumulation of autophagosomes, failed to stimulate the secretion of TDP-43. Therefore, specific pathways stimulated by Baf/Con are required for the release of autophagy-associated vesicles from cells.

The insufficiency of PGRN increased cleaved TDP-43 and LC3-II levels in the EV fraction, partially mimicking the effects of Baf/Con. We previously reported that the PGRN insufficiency and Baf treatment facilitated the nuclear translocation of TFEB (29, 35), which was linked to TDP-43 secretion in the present study. Further studies are needed to elucidate the mechanism of TDP-43 secretion regulated by PGRN and TFEB.

In affected areas of ALS and FTLD-TDP patients, TDP-43 accumulation in the cytoplasm is increased, resulting in the loss of function of TDP-43 in the nucleus (65). The present results indicated that the loss of function of TDP-43, mimicked by siRNA knockdown, promoted release of autophagy-associated vesicles when the autophagy-lysosome pathway was inhibited. Some of the causal genes for ALS or FTLD-TDP regulate the autophagy-lysosome pathway (21, 22, 66), and the inhibition of the autophagy-lysosome pathway in the sporadic ALS patients has been suggested (20). Thus, in the affected regions of ALS or FTLD-TDP patients, both the loss of function of TDP-43 and the inhibition of the autophagy-lysosome pathway might accelerate the cell-to-cell spread of pathological TDP-43 by the secretion of TDP-43 *via* autophagy-associated vesicles.

The aggregate-prone C-terminal domain of TDP-43 was necessary for Baf-induced secretion. The C-terminal fragment of TDP-43 lacks the nuclear localization signal and forms ubiquitin-positive inclusion-like structures that undergo pathological phosphorylation (50). On the other hand, the N-terminal domain is essential for nuclear localization and is negative for ubiquitination (50, 67). Upon the Baf treatment, the C-terminal fragment of TDP-43 localized within autophagosomes. This result is consistent with previous findings showing the autophagy-induced degradation of the C-terminal fragment of GFP-TDP-43 (29). Tanji *et al*. (68) reported that the autophagic adaptor p62, which was enriched in the P1 and P2 fractions, bound to TDP-43 by a ubiquitin-associated mechanism, and the upregulation of p62 facilitated the degradation of cleaved TDP-43. p62 plays a key role in promoting the incorporation of ubiquitinated proteins into autophagosomes (69). Therefore, the incorporation of the TDP-43 C-terminal domain by a ubiquitin-associated mechanism into autophagy-associated vesicles may drive the secretion of TDP-43.

The mechanisms by which TDP-43 inclusions in the cytoplasm contribute to neurodegeneration currently remain unclear. However, Cascella *et al*. (27) showed a close correlation between neuronal dysfunction and the presence of large-sized TDP-43 inclusions, which should be degraded through autophagy. The inhibition of the autophagy-lysosome pathway may result in the accumulation and secretion of neurotoxic TDP-43 inclusions.

In conclusion, the present study provides new lines of evidence for the mechanisms underlying the cell-to-cell spread of pathological TDP-43, which is suppressed by the PGRN-associated autophagy-lysosomal pathway. Higher TDP-43 levels in the CSF of ALS and FTLD-TDP patients (12, 13) may reflect TDP-43 secretion *via* autophagy-associated vesicles through an impaired autophagy-lysosomal pathway (70). To elucidate the pathoetiology of TDP-43 proteinopathy, a more detailed understanding is needed of the molecular basis of TDP-43 secretion upon the dysfunction of the autophagy-lysosomal pathway. In this study, we employed agonists and antagonists of autophagy as research tools triggering EV release. Further investigation is requited to ascertain the extent to which these pharmacologically induced effects accurately reflect the pathophysiological conditions that underlie the etiology associated with ALS or FTLD-TDP. We also need to clarify the mechanisms by which PGRN regulates the autophagy-lysosomal pathway, which is crucial for the development of treatment strategies for TDP-43 proteinopathy.

## Experimental procedures

### Antibodies and reagents

A monoclonal mouse anti-TDP-43 antibody (catalog no.: 60019-2-AP, Research Resource Identifier [RRID]: AB_2200520), polyclonal rabbit anti-C-terminal TDP-43 antibody (catalog no.: 12892-1-AP, RRID: AB_2200505), polyclonal rabbit anti-N-terminal TDP-43 antibody (catalog no.: 10782-2-AP, RRID: AB_615042), polyclonal rabbit anti-PGRN antibody (catalog no.: 18410-1-AP, RRID: AB_10598161), polyclonal rabbit anti-TSG101 antibody (catalog no.: 28283-1-AP, RRID: AB_2881104; catalog no.: 14497-1-AP, RRID: AB_2208090), polyclonal rabbit anti-ACTN4 antibody (catalog no.: 19096-1-AP, RRID: AB_10642150), polyclonal rabbit anti-ADAM10 antibody (catalog no.: 25900-1-AP, RRID: AB_2880291), and polyclonal rabbit anti-TFEB antibody (catalog no.: 13372-1-AP, RRID: AB_2919794) were purchased from ProteinTech; a monoclonal mouse anti-α-tubulin antibody (catalog no.: 70-102) and polyclonal rabbit anti-GFP antibody (catalog no.: 60-011) from BioAcademia; a monoclonal mouse anti-LC3 antibody (catalog no.: M186-3), normal rabbit immunoglobulin G (IgG) (catalog no.: PM035), polyclonal rabbit anti-Syntaxin-17 (human) antibody (catalog no.: PM076), and polyclonal rabbit anti-ATG16L1 antibody (catalog no.: PM040, RRID: AB_1278757) from MBL; a monoclonal mouse anti-p62 antibody (catalog no.: 610832, RRID: AB_398151) from BD Biosciences; a polyclonal sheep antimouse PGRN antibody (catalog no.: AF2557, RRID: AB_2114504) from R&D Systems; a monoclonal mouse anti-WIPI2 antibody (catalog no.: MCA5780GA, RRID: AB_10845951) from Bio-Rad; a biotin-conjugated horse antimouse IgG antibody (catalog no.: BA-2000, RRID: AB_2313581), biotin-conjugated goat anti-rabbit IgG antibody (catalog no.: BA-1000, RRID: AB_2313606), and biotin-conjugated rabbit antisheep IgG antibody (catalog no.: BA-6000, RRID: AB_2336217) from Vector Laboratories; and Alexa Fluor 568-conjugated donkey antimouse IgG (catalog no.: ab175472, RRID: AB_2636996), and Alexa Fluor 488–conjugated donkey antisheep IgG (catalog no.: ab150177, RRID: AB_2801320) from Abcam.

Hoechst 33342 (catalog no.: H342), DALG (catalog no.: D675), PlasMem Bright Green (catalog no.: P504), and PlasMem Bright Red (catalog no.: P505) were purchased from Dojindo; Baf (catalog no.: BVT-0252), Con (catalog no.: BVT-0237), and Rapa (catalog no.: AG-CN2-0025) were purchased from Adipogen; 4′,6-diamidino-2-phenylindole (DAPI) solution (catalog no.: 19178-91) and dimethyl sulfoxide (DMSO) (catalog no.: 09659-14) were purchased from Nacalai Tesque; E64d (4321-v) and pepstatin A (4397-v) were purchased from the Peptide Institute; AcidiFluor ORANGE (GC301) was purchased from Goryo Chemical; CQ diphosphate (catalog no.: 038-17971) was purchased from Fujifilm Wako; Vac (catalog no.: 20425) was purchased from Cayman Chemical; MG (catalog no.: BML-PI102) was purchased from Enzo Life Sciences; and PNGase F (catalog no.: P0704) was purchased from New England Biolabs.

### Cell culture

HeLa cells (CCL-2, American Type Culture Collection, RRID: CVCL_0030), HeLa cells stably expressing mRFP-GFP-LC3 (71, 72), ATG16L1 (−/−) HeLa cells (51), and SH-SY5Y cells (CRL-2266, American Type Culture Collection) were kindly gifted by Dr David Rubinsztein’s Lab (Cambridge Institute for Medical Research). HeLa cells stably expressing GFP-LC3-RFP-LC3ΔG (45) were kindly gifted by Dr Noboru Mizushima’s Lab. NSC-34 cells (CLU140) were purchased from CELLutions Biosystems. HeLa cells were cultured in Dulbecco’s modified Eagle’s medium (DMEM) with high glucose (catalog no.: 08458-45; Nacalai Tesque) containing 10% fetal calf serum and 1% penicillin–streptomycin (catalog no.: 26253-84; Nacalai Tesque). SH-SY5Y cells were cultured in DMEM/Ham’s F-12 (catalog no.: 08460-95; Nacalai Tesque) containing 10% fetal calf serum and 1% penicillin–streptomycin (catalog no.: 26253-84; Nacalai Tesque). NSC-34 cells were cultured in DMEM (catalog no.: D5796; Sigma–Aldrich) containing 10% fetal calf serum and 1% penicillin–streptomycin (catalog no.: 26253-84; Nacalai Tesque). Cells were maintained at 37 °C in the presence of 5% (v/v) CO_2_, and the number of passages was within 20 because a new vial was thawed. Mycoplasma contamination was tested using EZ-PCR (Sartorius).

### Quantification of autophagosome and autolysosome numbers

HeLa cells stably expressing mRFP-GFP-LC3 (71, 72) on coverslips (Matsunami Glass) cultured under the indicated conditions were fixed for 20 min with 4% paraformaldehyde in PBS (Nacalai Tesque) and subjected to confocal microscopy. The total numbers of GFP–RFP (autophagosome)- and RFP (autolysosome)-positive puncta per cell were counted using ImageJ (National Institutes of Health) with the Analyse Particles plugin (a constant threshold for all images per experiment was applied). At least 20 cells in each of the three independent experiments were subjected to counting.

### Assessment of autophagic flux

HeLa cells stably expressing GFP-LC3-RFP-LC3ΔG seeded at a density of 0.5 × 10^5^ cells on 12-well plates cultured under the indicated conditions for 24 h were lysed in 1% (w/v) Sarkosyl in A68 (10 mM Tris–HCl buffer, pH 7.5, 0.8 M NaCl, and 1 mM ethylene glycol bis(β-aminoethyl ether)*-N,N,N,N*-tetraacetic acid). Protein concentrations in the lysates were estimated using a BCA Protein Assay Kit (Thermo Fisher Scientific), and SDS-sample buffer was added to give a final concentration of 1% SDS. Samples were boiled for 10 min and subjected to SDS-PAGE followed by an immunoblot analysis to quantify GFP and RFP levels.

### Immunoblot analysis

Immunoblotting was performed as previously reported (29, 67). Samples were separated by SDS-PAGE and transferred onto polyvinylidene difluoride membranes (Millipore). Blots were blocked with Blocking One (Nacalai Tesque) and incubated overnight with the indicated primary antibody in Tris-buffer containing 10% (v/v) calf serum at room temperature (RT). Membranes were washed and incubated with a biotin-conjugated secondary antibody (Vector Laboratories) at RT for 2 h. To form the avidin–biotin complex, membranes were incubated with 0.4% (v/v) solutions A and B from the ABC standard kit (catalog no.: PK-4000; Vector Laboratories) at RT for 1 h. Colorimetric detection was conducted using PBS containing 0.04% (w/v) diaminobenzidine (Nacalai Tesque), 0.8% (w/v) NiCl·6H_2_O (Nacalai Tesque), and 0.3% (v/v) H_2_O_2_ (FUJIFILM Wako). Images were digitized by a scanner and subjected to densitometry using ImageJ. To quantify cleaved TDP-43 bands, four cleaved TDP-43 bands surrounded by the dashed square were subjected to densitometry. The specificity of each antibody used in immunoblot analysis was validated based on the molecular weight.

### Immunocytochemistry

Immunocytochemistry was carried out as shown previously (67). Cells grown on coverslips were fixed with 4% paraformaldehyde in PBS for 20 min and permeabilized with 0.3% (v/v) Triton X-100 in PBS (0.3% PBST) for 10 min. After blocking with 0.3% PBST containing 5% bovine serum albumin (Sigma–Aldrich) for 30 min, cells were incubated with the primary antibody at 37 ºC for 2 h. Cells were washed with 0.03% PBST and further incubated with the Alexa 488- or 568-conjugated secondary antibody at 37 ºC for 2 h. Cells were incubated with 0.03% PBST containing DAPI solution (1:3000 dilution) for nuclear staining and then washed with 0.03% PBST. Coverslips were sealed with VECTASHIELD Mounting Medium with DAPI (catalog no.: H-1200; Vector Laboratories). Samples were analyzed using an LSM780 confocal laser microscope (Carl Zeiss). The specificity of each antibody used in immunocytochemistry was validated based on the cellular localization. In addition, cells cultured in the SS condition were used as a positive control for WIPI2 immunostaining. PGRN KO MEFs were used as a negative control for PGRN immunostaining.

### Assessment of phagophore formation

As described in a previous study (29), HeLa cells seeded at a density of 0.5 × 10^5^ cells on coverslips (Matsunami Glass) and cultured under the indicated conditions for 24 h were fixed for 20 min with 4% paraformaldehyde in PBS and subjected to WIPI2 immunostaining to assess phagophore formation. WIPI2-positive puncta were monitored using the LSM780 confocal laser microscope equipped with a 60× objective lens. The signal intensity per cell was quantified using ImageJ and the Analyse Particles plugin (a constant threshold for all images per experiment was applied). At least 33 cells in each of the three independent experiments were subjected to counting.

### Assessment of the inhibition of lysosomal degradation on LC3-II levels

As described in a previous study (73), HeLa cells seeded at a density of 0.5 × 10^5^ cells on a 12-well plate cultured under the indicated conditions with or without 10 μg pepstatin A and E64d for 24 h were lysed in 1% (w/v) Sarkosyl in A68. Protein concentrations in the lysates were estimated using a BCA Protein Assay Kit, and SDS-sample buffer was added to give a final concentration of 1% SDS. Samples were boiled for 10 min and subjected to SDS-PAGE followed by immunoblotting to quantify LC3-II levels.

### Assessment of the acidity of lysosomes

HeLa cells in 12-well plates were incubated with AcidiFluor ORANGE for 2 h under the indicated conditions and subjected to live-cell imaging using the BZ-X710 fluorescence microscope (Keyence) equipped with a 20× objective lens. Five different frames of images (>335 cells) were captured, and the AcidiFluor ORANGE signal intensity per image was quantified using ImageJ with the Analyse Particles plugin (a constant threshold for all images per experiment was applied). The signal intensity per cell was calculated from the total value of the signal intensity divided by the total number of cells counted from Hoechst 33342 staining. Three independent experiments were performed for quantification.

### Preparation of cellular and EV fractions

Cellular and EV fractions were prepared using the method reported by Tripisciano *et al*. (74). To prepare the cellular fraction, cells were washed with cold PBS, detached from the dish by trypsinization, collected in a 15 ml tube, and centrifuged at 3000*g* at 4 °C for 10 min. The supernatant was discarded, and cells were lysed with 1% (w/v) Sarkosyl in A68 by sonication. Protein concentrations in cell lysates were estimated using a BCA Protein Assay Kit, and SDS-sample buffer was added to give a final concentration of 1% SDS. Regarding the fractionation of EV in culture medium, culture medium collected from the 10 cm dish was precleared by centrifugation at 800*g* at 4 °C for 10 min to remove nuclei and cell debris. The precleared medium was pooled in a centrifugation tube (catalog no.: 361706; Beckman Coulter) and centrifuged at 20,000*g* at 4 °C for 1 h. The pellet was collected as the P1 fraction and lysed with 50 μl 2% SDS-sample buffer. Supernatants were recollected in the centrifugation tube (catalog no.: 344059; Beckman Coulter) and centrifuged at 110,000*g* at 4 °C for 1 h. The pellet was collected as the P2 fraction and lysed with 50 μl of 2% SDS-sample buffer. Samples were boiled for 10 min and subjected to an immunoblot analysis.

### Immunoprecipitation

Immunoprecipitation was performed as previously described (67). Briefly, the cell, P1, and P2 fractions prepared from HeLa cells exposed to 100 nM Baf for 24 h were lysed with 150 μl radioimmunoprecipitation assay buffer (Nacalai Tesque) and subjected to immunoprecipitation with normal rabbit IgG or the polyclonal rabbit anti-C-terminal TDP-43 antibody (5 μl) and protein G-Sepharose (5 μl, Sigma–Aldrich). Bound proteins were washed with radioimmunoprecipitation assay buffer three times and then eluted from the beads with SDS-sample buffer. Each sample was separated by SDS-PAGE and immunoblotted with the mouse monoclonal anti-TDP-43 antibody.

### Immunoelectron microscopy

Immunoelectron microscopy was conducted as previously described (9). The P1 and P2 fractions derived from HeLa cells treated with DMSO or 100 nM Baf were suspended in 20 μl PBS. Samples of between 1 and 2 μl were placed onto carbon-coated 300-mesh copper grids (Nissin EM), blocked with 0.3% gelatin in PBS, and incubated with the primary antibody to C-terminal TDP-43 (ProteinTech) at a dilution of 1:100 in 0.3% gelatin in PBS at 37 ºC for 1 to 2 h. After washing with PBS, the grids were incubated with secondary antibodies conjugated to 10 nm gold particles (Cytodiagnostics, Inc) at a dilution of 1:50 in 0.3% gelatin in PBS at 37 ºC for 1 h. The grids were then stained with 2% phosphotungstate. Electron micrograph images were acquired using a JEOL JE<-1400 electron microscope operated at 80 keV and equipped with a CCD camera.

### LC–ion trap mass spectrometry (LC–MS/MS) analysis

LC–MS/MS was performed according to our previously reported protocol (75). The P1 and P2 fractions prepared from HeLa cells exposed to 100 nM Baf were suspended in 30 μl PBS and mixed with 10 μl of 200 ng/μl trypsin solution. After digestion at 37 ºC for 20 h, 5 μl of 100 mM dithiothreitol was added. The mixture was then incubated at 95 ºC for 5 min and dried using Speed Vac (Thermo Scientific, Inc). In the LC–MS/MS analysis, digests were dissolved into 50 μl of 0.1% trifluoroacetic acid and introduced into the nano-flow HPLC system, EASY-nLC 1200 (Thermo Fisher Scientific, Inc). The packed nanocapillary column NTCC-360/75-3-123 (0.075 mm I.D. × 125 mm l, particle diameter 3 μm; Nikkyo Technos Co, Ltd) was used at a flow rate of 300 nl/min with a 2 to 80% linear gradient of acetonitrile for 80 min. Eluted peptides were directly detected with the ion trap mass spectrometer, QExactive HF (Thermo Fisher Scientific, Inc). A spray voltage of 2.0 kV and capillary temperature of 250 °C was used for ionization. The mass acquisition method consisted of one full MS survey scan with an Orbitrap resolution of 60,000 followed by an MS/MS scan of the most abundant precursor ions from the survey scan with an Orbitrap resolution of 15,000. Dynamic exclusion for MS/MS was set to 30 s. An MS scan range of 350 to 1800 *m/z* was employed in the positive ion mode, followed by data-dependent MS/MS using the higher-energy collisional dissociation operating mode on the top 15 ions in order of abundance. Data were analyzed with Proteome Discoverer (version 2.4; Thermo Fisher Scientific, Inc), Mascot software (version 2.5.1; Matrix Science, Inc), and Scaffold software (version 3; Proteome Software, Inc). The SwissProt database (https://www.uniprot.org/) updated on July 15, 2022 and GenBank database (https://www.ncbi.nlm.nih.gov/) updated on February 27, 2020 were searched with the following parameters: monoisotopic mass, peptide mass tolerance of 10 ppm, fragment ion mass tolerance of 0.05 Da, complete tryptic digestion allowing three missed cleavages, static modification of beta-methylthiolation, variable modifications of methionine oxidation, deamidation, and pyroglutamination. A scaffold was used for label-free quantification. Peptide and protein probability thresholds of 99 and 99%, respectively, were applied. Using a target decoy approach, a peptide false discovery rate of 0.1% was selected. Data are presented in Dataset S4 (the P1 fraction) and Dataset S5 (the P2 fraction).

### Cellular component analysis

Proteins detected by the LC–MS/MS analysis were divided into three groups: proteins detected in the P1 fraction only, in the P2 fraction only, and in both the P1 and P2 fractions. The cellular components of proteins in these groups were examined using DAVID software (48).

### Expression vectors

mCherry-hLC3B-pcDNA3.1 (catalog no.: 40827, from Rubinsztein’s Lab) was purchased from Addgene. Full-length TDP-43 (amino acids 1–414) in EGFPC1 (Clontech), the C-terminal fragment of TDP-43 (amino acids 162–414) in EGFPC1, and the N-terminal fragment of TDP-43 (amino acids 1–273) in EGFPC1 were previously characterized (50, 67).

### Generation of HeLa cells stably expressing mCherry-LC3

To generate HeLa cells stably expressing mCherry-LC3, HeLa cells seeded on a 6 cm dish were transfected with 1 μg of a mCherry-LC3 construct and 3 μl of TransIT2020 reagent (Mirus) according to the manufacturer’s protocol. G418 (600 μg/ml; FUJIFILM Wako)-resistant clones stably expressing mCherry-LC3 were obtained by limiting dilution cloning in 96-well plates. Steady-state expression levels were confirmed by immunoblotting.

### Transfection

Transfection was performed as previously reported (41). In transient expression experiments, cells were grown to 30 to 40% confluence in 3.5 or 6 cm dishes. They were then transfected with expression vectors using TransIT-2020 reagent (catalog no.: MIR5400; Mirus) according to the manufacturer’s instructions. In knockdown experiments, cells were transfected with 40 pmol siRNA using Lipofectamine RNAiMAX (catalog no.: 13778; Invitrogen). Predesigned stealth siRNA duplexes (Thermo Fisher Scientific) to target human *TARDBP* (HSS118766 [siTARDBP#1]: forward, 5′-GAC AGA UGC UUC AUC AGC AGU GAA A-3’; reverse, 5′-UUU CAC UGC UGA UGA AGC AUC UGU C-3′, and HSS104450 [siTARDBP#2]: forward, 5′-UGA GCC CAU UGA AAU ACC AUC GGA A-3’; reverse, 5′-UUC CGA UGG UAU UUC AAU GGG CUC A-3′), human *STX17* (HSS123732: forward, 5′-GGA AAC CUU AGA AGC GGA CUU AAU U-3’; reverse, 5′-AAU UAA GUC CGC UUC UAA GGU UUC C-3′), human *TFEB* (HSS111870: forward, 5′-UGA AAU GCA GAU GCC CAA CAC GCU A-3’; reverse, 5′-UAG CGU GUU GGG CAU CUG CAU UUC A-3′), mouse *Grn* (MSS204933: forward, 5′-GGA ACC AAG UGU UUG CGA AAG AAG A-3’; reverse, 5′-UCU UCU UUC GCA AAC ACU UGG UUC C-3′), were used for the knockdown of human *TARDBP*, *STX17*, *TFEB*, and mouse *Grn*, respectively. Predesigned stealth siRNA duplexes to target human *GRN* (HSS104450: forward, 5′-AGA UCG UGG CUG GAC UGG AGA AGA U-3’; reverse, 5′-AUC UUC UCC AGU CCA GCC ACG AUC U-3′) were used for the knockdown of human *GRN*, as previously reported (37, 41). The stealth RNAi Negative Control Duplex, Med GC (catalog no.: 12935300; Thermo Fisher Scientific) was used as a negative control of siRNA.

### The ratio of GFP-tagged full-length, N-terminal, or C-terminal TDP-43 colocalized with mCherry-LC3 under the Baf treatment

GFP-tagged expression vectors of full-length (amino acids 1–414), the N-terminal fragment (amino acids 1–273), and C-terminal fragment (amino acids 162–414) of TDP-43 were previously prepared (50, 67). HeLa cells stably expressing mCherry-LC3 were transfected for 24 h with the vectors and were then reseeded on μ-Dish, 35 mm high (ibidi). Cells were cultured in complete medium containing 100 nM Baf for 24 h and were then monitored in complete medium without phenol red (catalog no.: 08489-45; Nacalai Tesque) under the Baf condition using the 60× objective lens of the LSM780 confocal laser microscope. Each cell expressing the GFP-TDP-43 signal was subjected to measurements. After setting thresholds for the red (mCherry-LC3) and green (GFP-TDP-43) signals, the signal intensity of GFP-TDP-43 was calculated by the Analyse Particles plugin on Fiji (76). To measure the signal intensity of the double-positive area for GFP-TDP-43 and mCherry-LC3, green and red binary images were overlapped using the paste control “AND,” and signal intensity was calculated by the Analyse Particles plugin. The ratio of GFP-TDP-43 colocalized to mCherry-LC3 was calculated from the signal intensity double positive for green and red divided by the total green signal intensity. At least 20 cells in each of the three independent experiments were subjected to counting.

### GFP-tagged C-terminal TDP-43 (amino acids 162–414) and mCherry-LC3 localization on the plasma membrane

HeLa cells were transfected with TDP-43 amino acids 162 to 414 or mCherry-LC3 for 24 h and then seeded on μ-Dish, 35 mm high (ibidi). After a 24 h stimulation with 100 nM Baf, the plasma membrane was visualized with PlasMem Bright Green or PlasMem Bright Red according to the manufacturer’s protocol. Live-cell imaging was conducted using the LSM780 confocal laser microscope equipped with a 60× objective lens.

### Autolysosome analysis with DALG staining

As shown in previous studies (29, 67), autolysosome assays were conducted using DALG, which emits stronger green fluorescence in autolysosomes. DALG was loaded for 30 min on HeLa cells in μ-Slide 8 well high (ibidi). After washing, cells were treated for 2 h under the indicated conditions and subjected to live-cell imaging using the BZ-X710 fluorescence microscope. DALG signal intensity per cell was quantified using ImageJ with the Analyse Particles plugin (a constant threshold for all images per experiment was applied). At least 50 cells in each of the three independent experiments were subjected to counting.

### Generation of PGRN CRISPR KO mice

PGRN CRISPR KO mice were generated by improved-Genome editing *via* Oviductal Nucleic Acids Delivery (iGONAD) (77, 78). Briefly, 8- to 10-week-old C57BL/6 female mice were mated with male mice. The following day, mice under anesthesia were subjected to an injection into the oviduct of approximately 1 μl of solution containing 30 μM predesigned CRISPR RNA to target the mouse *Grn* gene (5′-GGCUACCAGCCCUGCCGCGAGUUUUAGAGCUAUGCUGUUUUG-3′, FASMAC), transactivating CRISPR RNA (5′AAACAGCAUAGCAAGUUAAAAUAAGGCUAGUCCGUUAUCAACUUGAAAAAGUGGCACCGAGUCGGUGCU-3′, FASMAC), and 1 mg/ml Cas9-NLS protein (NIPPON GENE), and electroporated using the NEPA21 electroporator (Nepagene) on embryonic day 0.7 (approximately 15:00). Newborn mice were weaned at 3 weeks old, and their genome was extracted from their tails. The alignment of the genome-edited site was identified with Sanger sequencing (Azent). Mice with the mutation p.Ala24GlyfsTer22 were selected and backcrossed with ordinary C57BL/6 mice three times. Mice were bred in the laboratory animal facility (Imabari) in accordance with the guidelines of the Institute for Laboratory Animal Research under controlled light (lights on, 07:00–19:00), temperature (23 ± 1 °C), and humidity (55 ± 10%), and were given free access to food and water. All animal experiments were performed in accordance with the Guide for the Care and Use of Laboratory Animals of Okayama University of Science and were approved by the Institutional Animal Care and Use Committee of Okayama University of Science (permit number: 2019-047).

### MEFs

The preparation of MEFs was described in a previous study (79). Embryos were derived from E13.5 PGRN WT and KO mouse embryos under anesthesia. The head above the eyes was cut off, and the heart and liver were removed from embryos. The remainder of the embryo was minced with scissors and dissociated with a strainer. Dissociated cell suspensions equivalent to three embryos were seeded onto 10 cm dishes. Cells that reached confluency were trypsinized and preserved with growth medium DMEM containing 10% DMSO in liquid nitrogen for later analyses.

### Deglycosylation of N-linked glycans

According to the manufacturer’s protocol, the N-linked glycans of proteins in the P1 and P2 fractions of WT MEFs were deglycosylated with PNGase F (catalog no.: P0704; New England Biolabs). Briefly, P1 and P2 pellets were prepared as shown previously. In the PNGase F-negative group (control), pellets were mixed with 2.5 μl GlycoBuffer 2 (catalog no.: P0704; New England Biolabs) and 22.5 μl H_2_O. In the PNGase F-positive group, pellets were mixed with 2.5 μl GlycoBuffer 2, 20 μl H_2_O, and 2.5 μl PNGase F. After a 24 h incubation at 37 °C, samples were mixed with 25 μl SDS-sample buffer (4% SDS, 30% glycerol, 10% 2-mercaptoethanol, 0.125 M Tris–HCl [pH 6.8], and 0.02% bromophenol blue) and boiled for 10 min for immunoblotting.

### Nuclear translocation of TFEB-GFP

SH-SY5Y cells stably expressing TFEB-GFP (29) seeded on the cover glass (Matsunami Glass) were exposed to the indicated drugs or transfected with the indicated siRNA. Cells were fixed and monitored using a 60× objective lens equipped to the LSM780 confocal laser microscope. The percentage of cells in which TFEB-GFP translocated in the nucleus per the total number of cells counted was calculated. At least 102 cells in each of the three independent experiments were subjected to counting.

### Statistical analysis

The unpaired two-tailed Student’s *t* test, Mann–Whitney *U* test, or Tukey’s post hoc test were performed using Excel (Microsoft Office) or EZR (80). Data are expressed as the mean ± SD. *p* < 0.05 was considered to be significant. The *p* values of all statistical comparisons between experimental groups are shown in Dataset S6.

## Data availability

All data are contained in the figures, figure legends, or supporting information.

## Supporting information

This article contains supporting information.

## Conflict of interest

The authors declare that they have no conflicts of interest with the contents of this article.
