# Supplementary material for: Dysregulation of the progranulin-driven autophagy-lysosomal pathway mediates secretion of the nuclear protein TDP-43
Source: J Biol Chem. 2023 Sep 20;299(11):105272. doi: 10.1016/j.jbc.2023.105272 (PMC10641265; doi:10.1016/j.jbc.2023.105272)
Supplement: Supporting information Tables [file mmc1.pptx]

## Slide 1
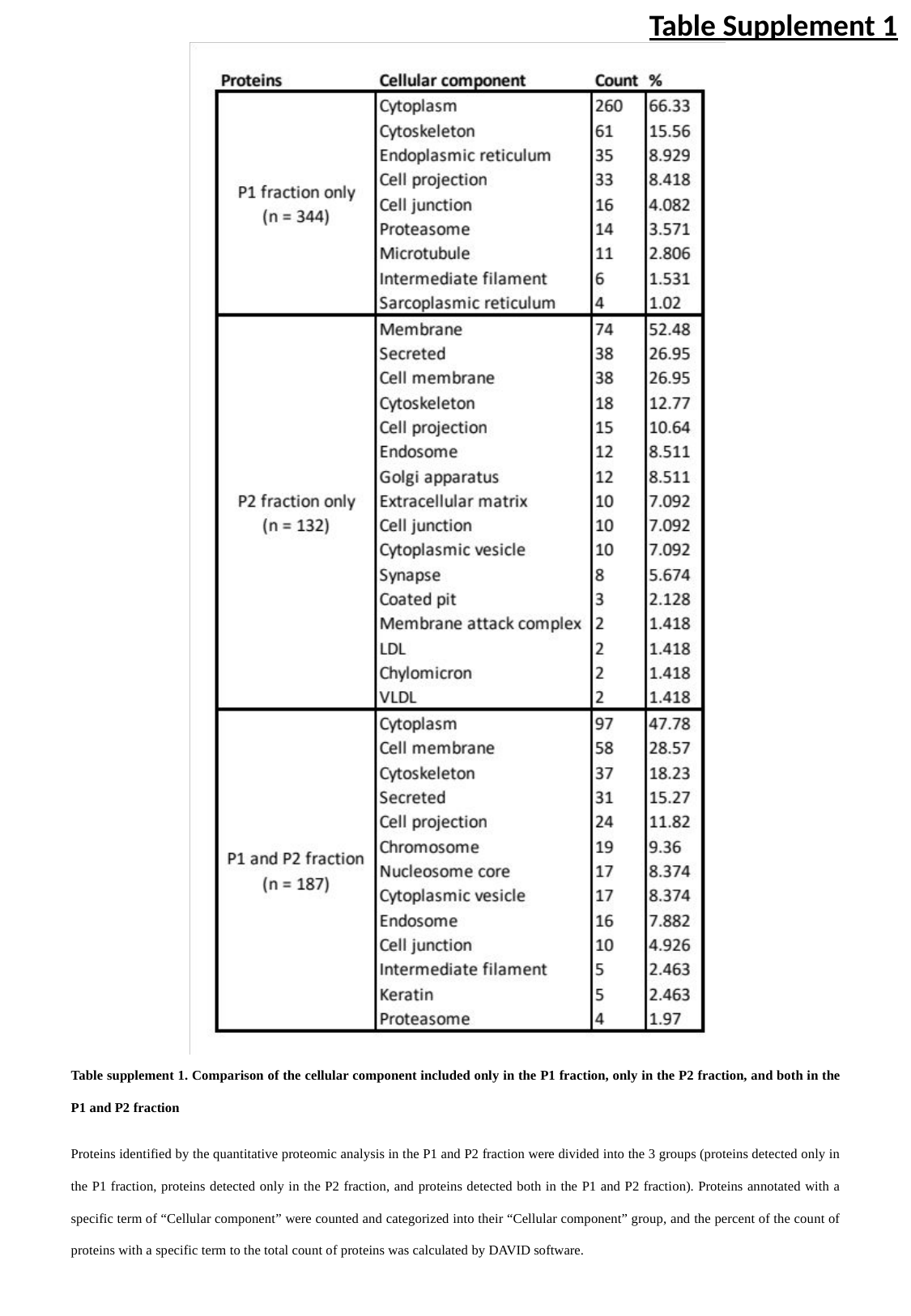

Table Supplement 1
Table supplement 1. Comparison of the cellular component included only in the P1 fraction, only in the P2 fraction, and both in the P1 and P2 fraction
Proteins identified by the quantitative proteomic analysis in the P1 and P2 fraction were divided into the 3 groups (proteins detected only in the P1 fraction, proteins detected only in the P2 fraction, and proteins detected both in the P1 and P2 fraction). Proteins annotated with a specific term of “Cellular component” were counted and categorized into their “Cellular component” group, and the percent of the count of proteins with a specific term to the total count of proteins was calculated by DAVID software.
