# Supplementary material for: Dysregulation of the progranulin-driven autophagy-lysosomal pathway mediates secretion of the nuclear protein TDP-43
Source: J Biol Chem. 2023 Sep 20;299(11):105272. doi: 10.1016/j.jbc.2023.105272 (PMC10641265; doi:10.1016/j.jbc.2023.105272)
Supplement: Supporting Figures [file mmc2.pdf]

Figure Supplement 1

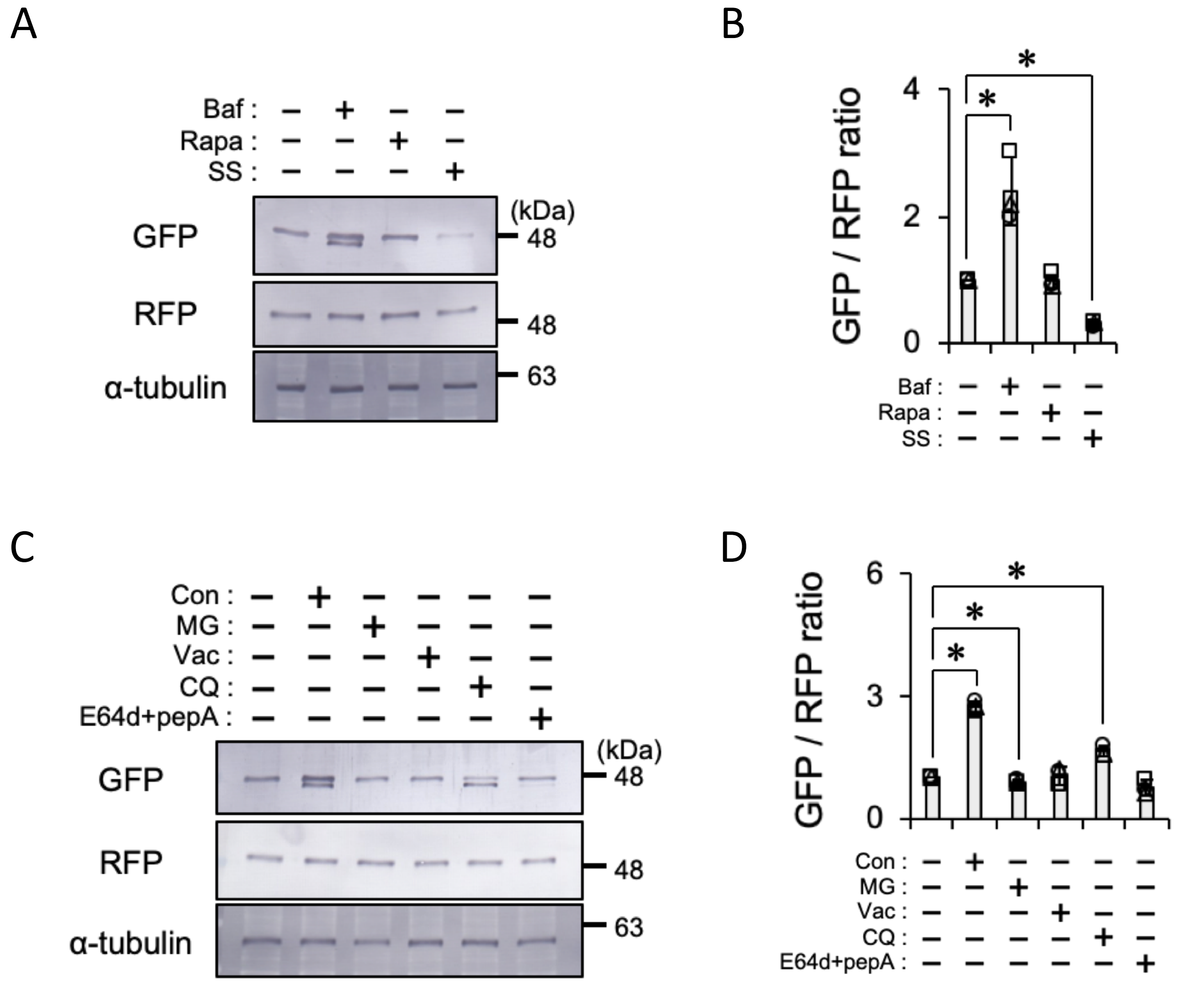

Figure supplement 1. The influence of drugs and serum starvation on autophagic flux in HeLa cells stably expressing GFP-LC3-RFP-LC3ΔG.

HeLa cells stably expressing GFP-LC3-RFP-LC3ΔG were treated with vehicle (DMSO), 100 nM Baf, 400 nM Rapa, serum starvation (SS), 100 nM Con, 1 μM MG, 100 nM Vac, 100 μM CQ, or 10 μg/mL E64d and pepstatin A (pepA) for 24 hrs. (**A, C**) Representative immunoblots (GFP, RFP, and α-tubulin) of cells exposed to indicated treatement are shown (**B, D**) The GFP/RFP ratio was calculated from densitometric data of GFP and RFP immunoblotting results. The GFP/RFP ratio normalized to cells exposed to vehicle are shown. In bar graphs, data are presented as means ± S.D. (N = 3). * indicates P <0.05 by the two-tailed unpaired t-test.

Figure Supplement 2

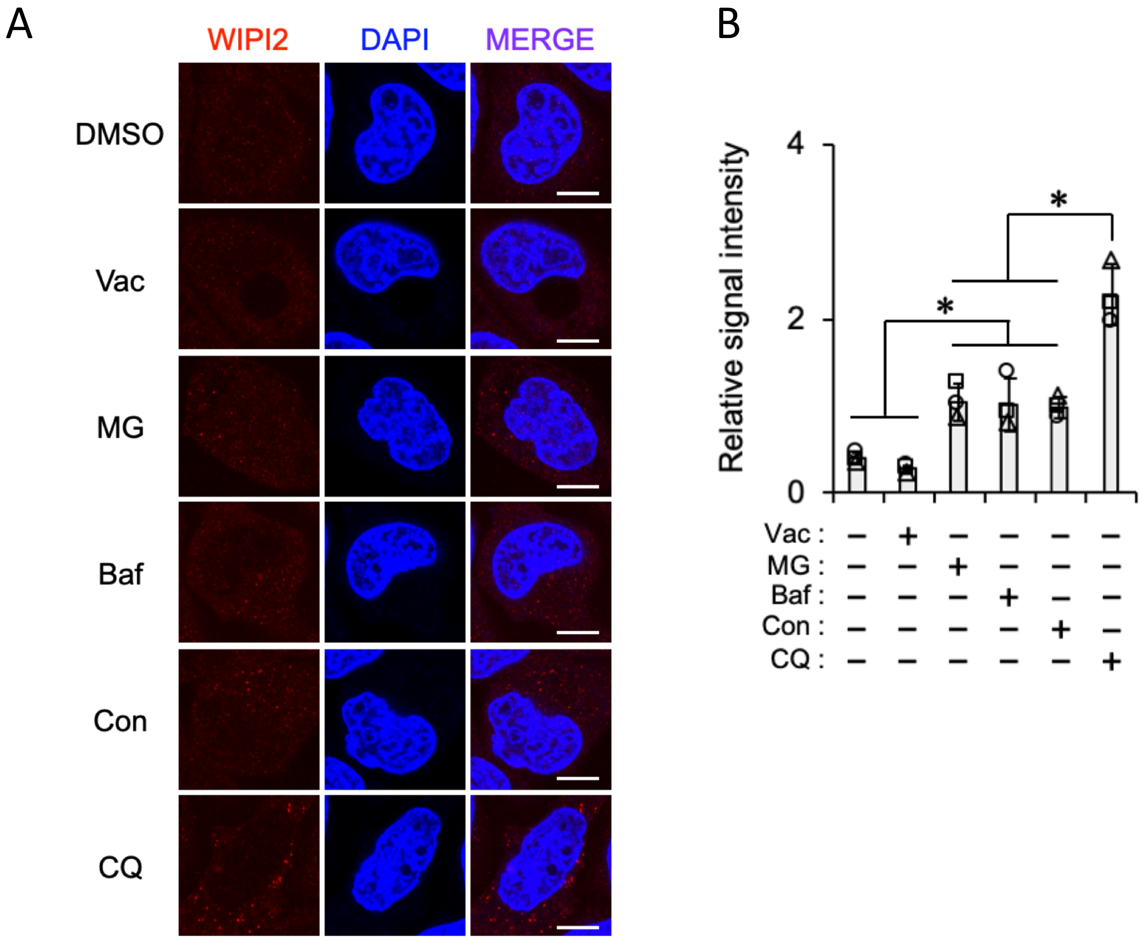

Figure supplement 2. The influence of drugs regulating autophagy on phagophore formation in HeLa cells

HeLa cells were exposed to vehicle (DMSO), 100 nM Vac, 1 μM MG, 100 nM Baf, 100 nM Con, or 100 μM CQ for 24 hrs. (**A**) Representative confocal images of WIPI2 immunostaining of cells treated with the indicated drug. Nuclei were stained with DAPI (Blue). Scale bar, 10 μm. (**B**) The relative signal intensity normalized to the average of signal intensity in each experiment are shown. In bar graphs, data are presented as means ± S.D. ($N = 3$). * indicates $P < 0.05$ by the two-tailed unpaired *t*-test.

Figure Supplement 3

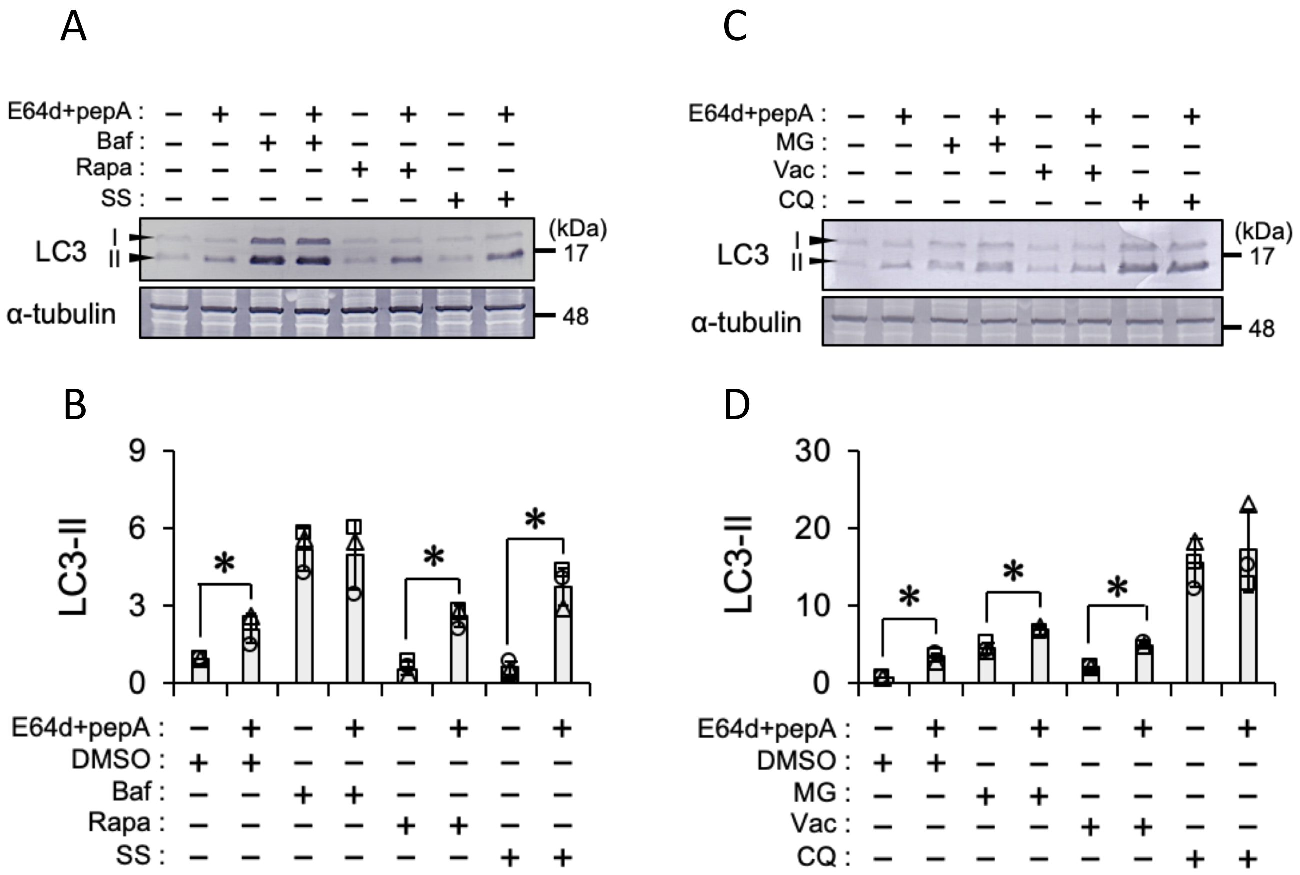

Figure supplement 3. The influence of inhibition of lysosomal degradation on LC3-II levels of cells exposed to the indicated treatment in HeLa cells.

HeLa cells were treated with vehicle (DMSO), 100 nM Baf, 400 nM Rapa, serum starvation (SS), 1 μM MG, 100 nM Vac, or 100 μM CQ in addition to vehicle or 10 μg/mL E64d and pepstatin A (pepA) for 24 hrs. (**A, C**) Representative immunoblots (LC3 and α-tubulin) of cells exposed to indicated treatment in addition to vehicle or E64d and pepA are shown. (**B, D**) Densitometric data on LC3-II in the cell exposed to the indicated treatment were calculated from immunoblotting results. The relative signal intensity normalized against cells exposed to vehicle only are shown. In bar graphs, data are presented as means ± S.D. ($N = 3$). * indicates $P < 0.05$ by the two-tailed unpaired t-test.

Figure Supplement 4

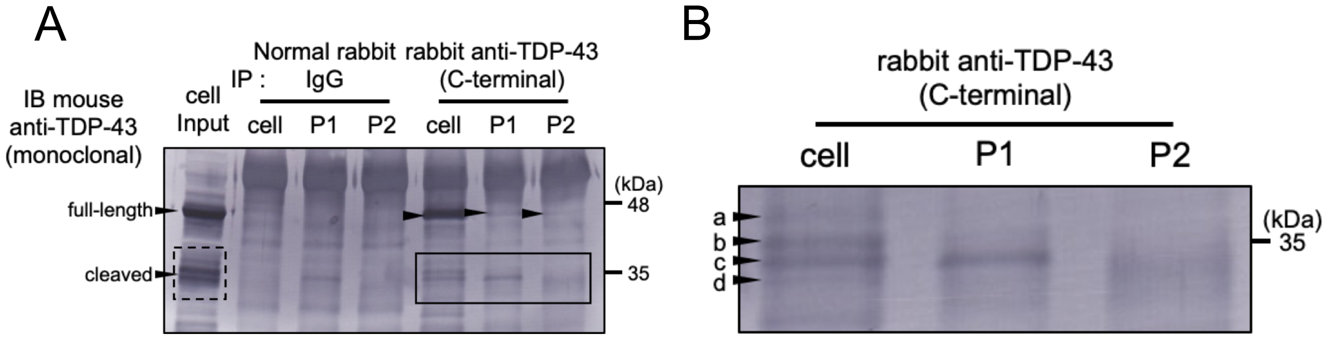

Figure supplement 4. Immunoprecipitation using the C-terminal TDP-43 antibody indicates the difference of cleaved TDP-43 band in the cell and EV fraction.

The cell, P1 (20,000 × *g* pellet), and P2 (110,000 × *g* pellet) fractions from HeLa cells exposed to 100 nM Baf for 24 hrs were immunoprecipitated with the normal rabbit IgG or rabbit anti-C-terminal TDP-43 antibody. The immunoprecipitated samples were immunoblotted with the mouse monoclonal anti-TDP-43 antibody. (**A**) The representative image of TDP-43 immunoblots detected by the mouse monoclonal anti-TDP-43 antibody are shown. The dashed square indicates bands of cleaved TDP-43 used for the quantification. The image surrounded by a black square was magnified in (**B**). Bands with a different letter (a, b, c, and d) indicates separate bands.

Figure Supplement 5

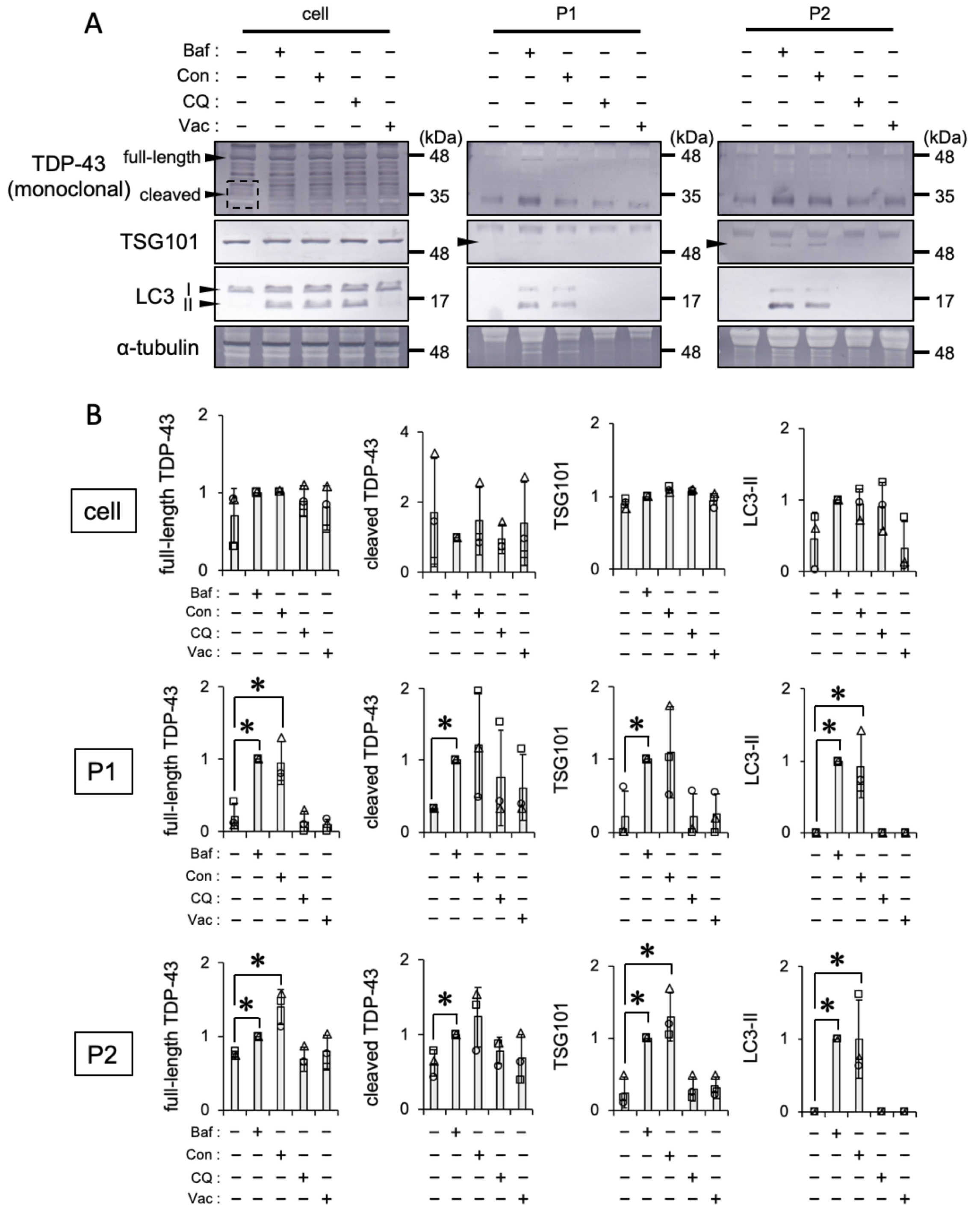

Figure supplement 5. Bafilomycin A1 and concanamycin A increases TDP-43, LC3-II, and TSG101 levels in the extracellular vesicle fraction in SH-SY5Y cells

SH-SY5Y cells were exposed to vehicle (DMSO), 100 nM Baf, 100 nM Con, 100 μM CQ, or 100 nM Vac for 24 hrs. The cell, P1 (20,000 × *g* pellet), and P2 (110,000 × *g* pellet) fractions in each treatment were prepared as shown in the Experimental Procedures. (**A**) Representative immunoblots (TDP-43 detected by the monoclonal antibody, TSG101, LC3, and α-tubulin) of each fraction are shown. The dashed square indicates bands of cleaved TDP-43 used for quantification. (**B**) Densitometric data on full-length TDP-43, cleaved TDP-43, TSG101, and LC3-II in the cell and EV fraction were calculated from immunoblotting results. The relative signal intensity normalized against cells exposed to Baf are shown. In bar graphs, data are presented as means ± S.D. ($N = 3$). * indicates $P < 0.05$ by the two-tailed unpaired *t*-test.

Figure Supplement 6

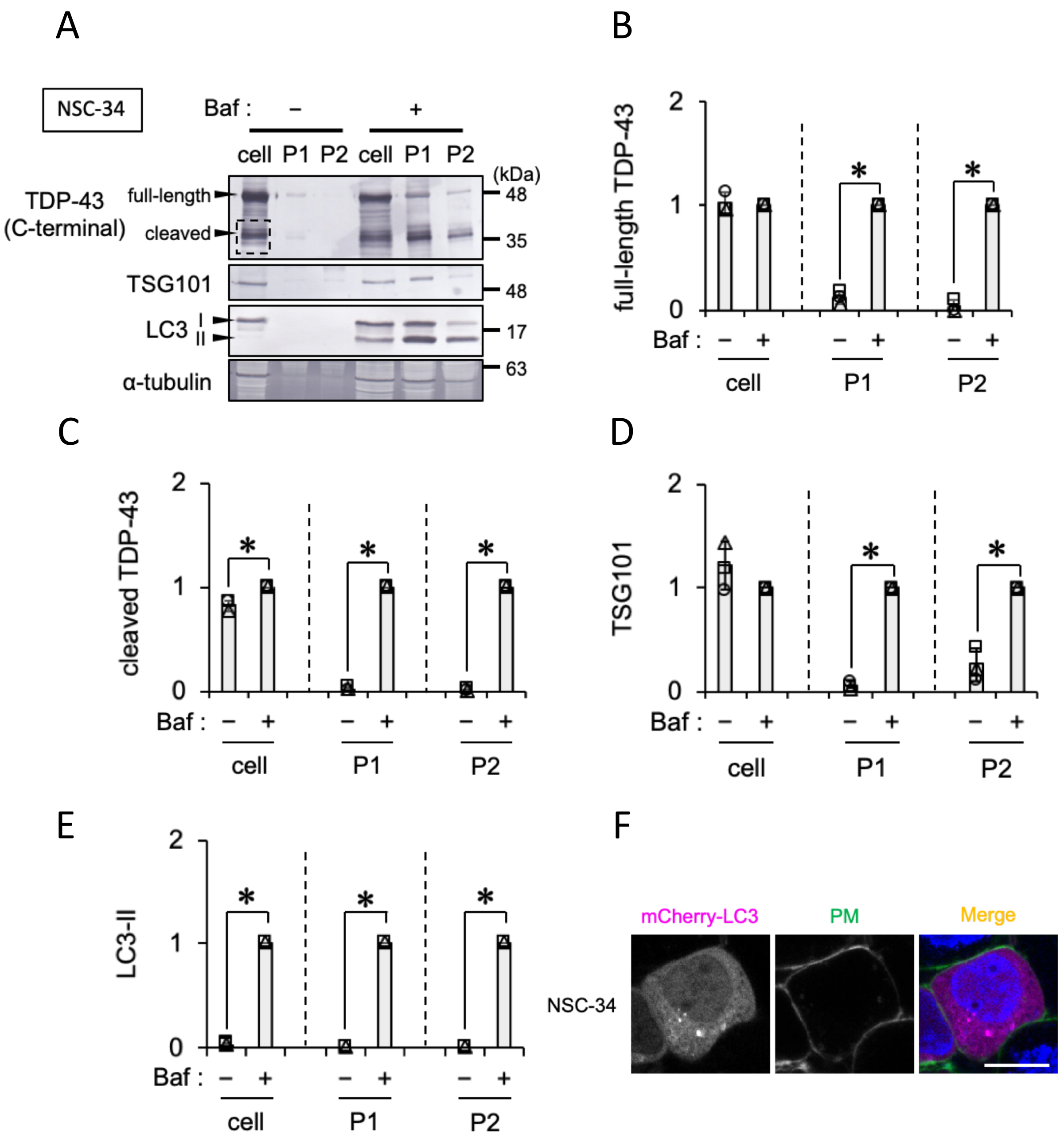

Figure supplement 6. Bafilomycin A1 increases TDP-43 and LC3-II levels in the extracellular vesicle fraction in NSC-34 cells

NSC-34 cells were exposed to vehicle (DMSO) or 100 nM Baf for 24 hrs. The cell, P1 (20,000 × *g* pellet), and P2 (110,000 × *g* pellet) fractions in each treatment were prepared as shown in the Experimental Procedures. (**A**) Representative immunoblots (TDP-43 detected by the polyclonal anti-C-terminal TDP-43 antibody, LC3, and α-tubulin) of each fraction are shown. The dashed square indicates bands of cleaved TDP-43 used for quantification. (**B-E**) Densitometric data on (**B**) full-length TDP-43, (**C**) cleaved TDP-43, (**D**) TSG101 and (**E**) LC3-II were calculated from immunoblotting results. The relative signal intensity normalized to cells exposed to Baf are shown. In bar graphs, data are presented as means ± S.D. (N = 3). * indicates P <0.05 by the two-tailed unpaired t-test. (**F**) Representative confocal images of live cells transfected with the mCherry-LC3 construct (Red) are shown. The PM was stained with PlasMem Bright Green (Green). Nuclei were stained with Hoechst33342. Scale bar, 10 µm.

Figure Supplement 7

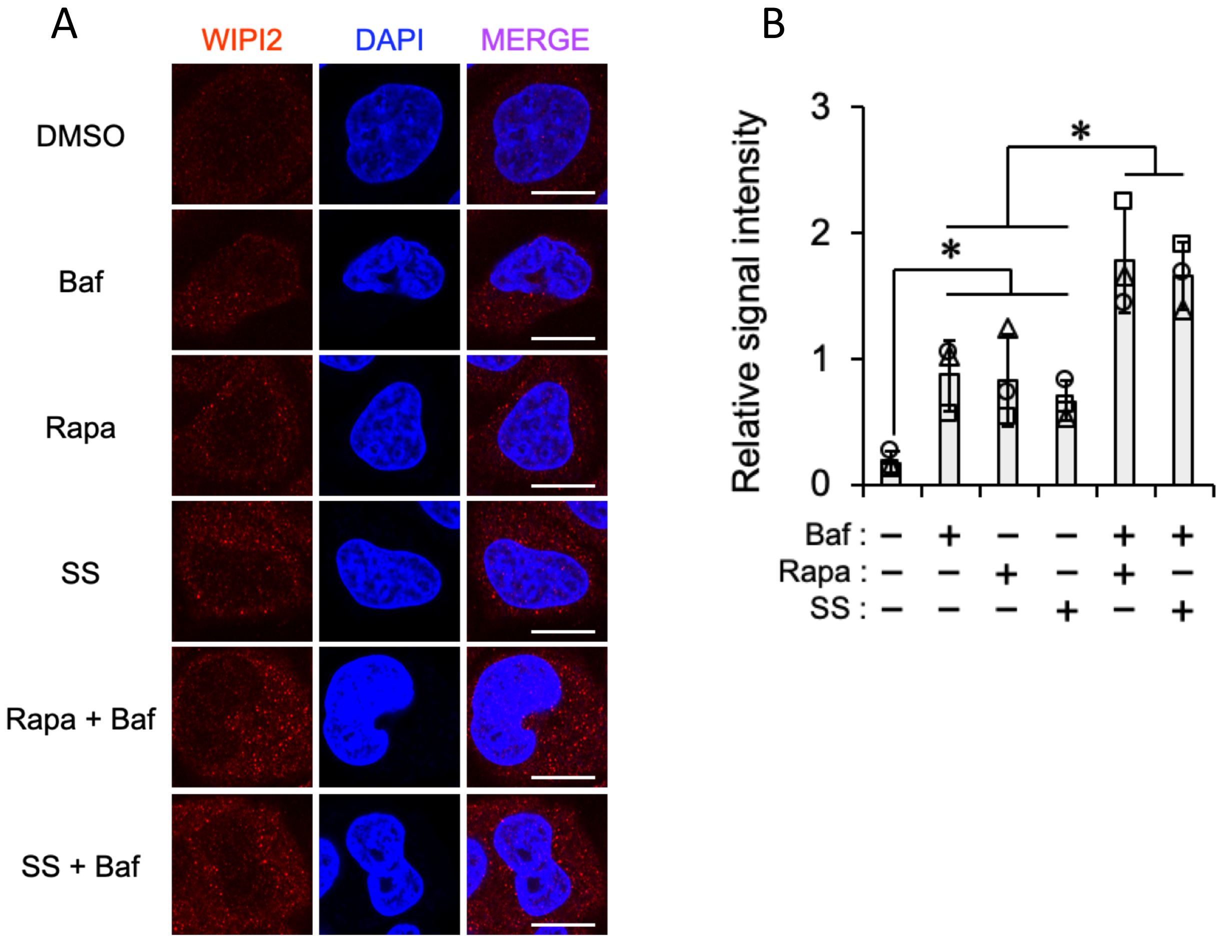

Figure supplement 7. The influence of the indicated treatment regulating autophagy on phagophore formation in HeLa cells

HeLa cells were treated with vehicle (DMSO), 100 nM Baf, 400 nM Rapa, serum starvation (SS), 400 nM Rapa plus 100 nM Baf, or SS plus 100 nM Baf for 24 hrs. (**A**) Representative confocal images of WIPI2 immunostaining of cells treated with the indicated treatment. Nuclei were stained with DAPI (Blue). Scale bar, 10 µm. (**B**) The relative signal intensity normalized to the average of signal intensity in each experiment are shown. In bar graphs, data are presented as means ± S.D. ($N = 3$). * indicates $P < 0.05$ by the two-tailed unpaired *t*-test.

Figure Supplement 8

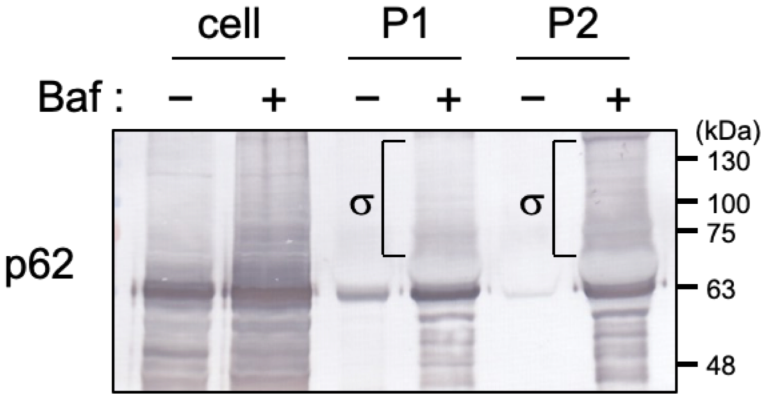

Figure supplement 8. Bafilomycin A1 increases p62 smeared bands in the cell and extracellular vesicle fraction in HeLa cells

HeLa cells were exposed to DMSO or 100 nM Baf for 24 hrs. The cell, P1 (20,000 × *g* pellet), and P2 (110,000 × *g* pellet) fractions in each treatment were prepared as shown in the Experimental Procedures. Representative p62 immunoblots of each fraction are shown. σ indicates smeared p62 bands in the P1 or P2 fraction derived from cells exposed to Baf.

Figure Supplement 9

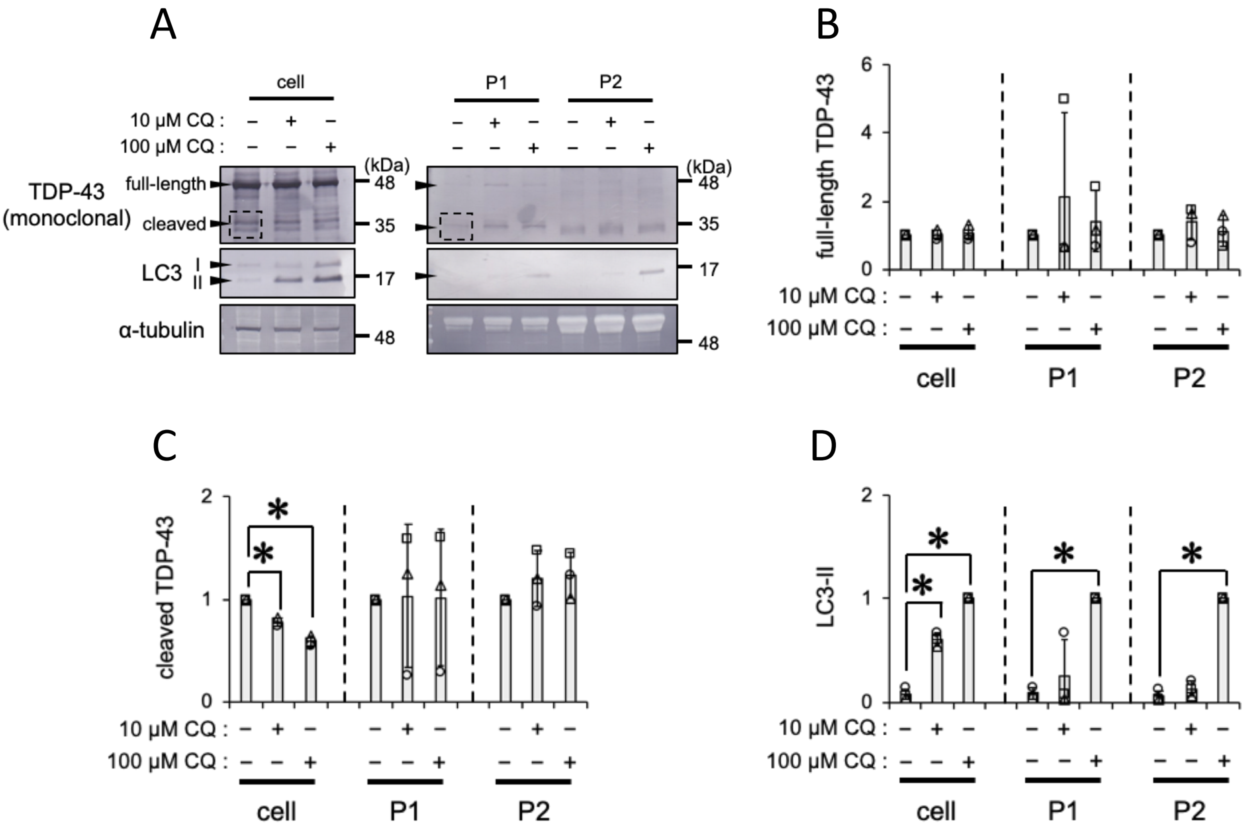

Figure supplement 9. Chloroquine dose-dependently increases LC3-II levels in the cell and EV fraction

(**A-D**) HeLa cells were exposed to 0 (distilled water), 10, and 100 μM chloroquine (CQ) for 24 hrs. Cellular fraction (cell) and EV fraction (P1; 20,000 × g pellet and P2; 110,000 × g pellet) in each treatment were prepared for immunoblotting as shown in the Experimental Procedures. (**A**) The representative immunoblots (TDP-43, LC3, and α-tubulin) of each fraction are shown. The dashed square indicates bands of cleaved TDP-43 used for quantification. (**B-D**) Densitometric data on (**B**) full-length TDP-43, (**C**) cleaved TDP-43, and (**D**) LC3-II in the cell and EV fraction were calculated from immunoblotting results. The relative signal intensity normalized to cells exposed to 0 or 100 μM CQ are shown. In bar graphs, data are presented as means ± S.D. ($N = 3$). * indicates $P < 0.05$ by two-tailed unpaired *t*-test.

Figure Supplement 10

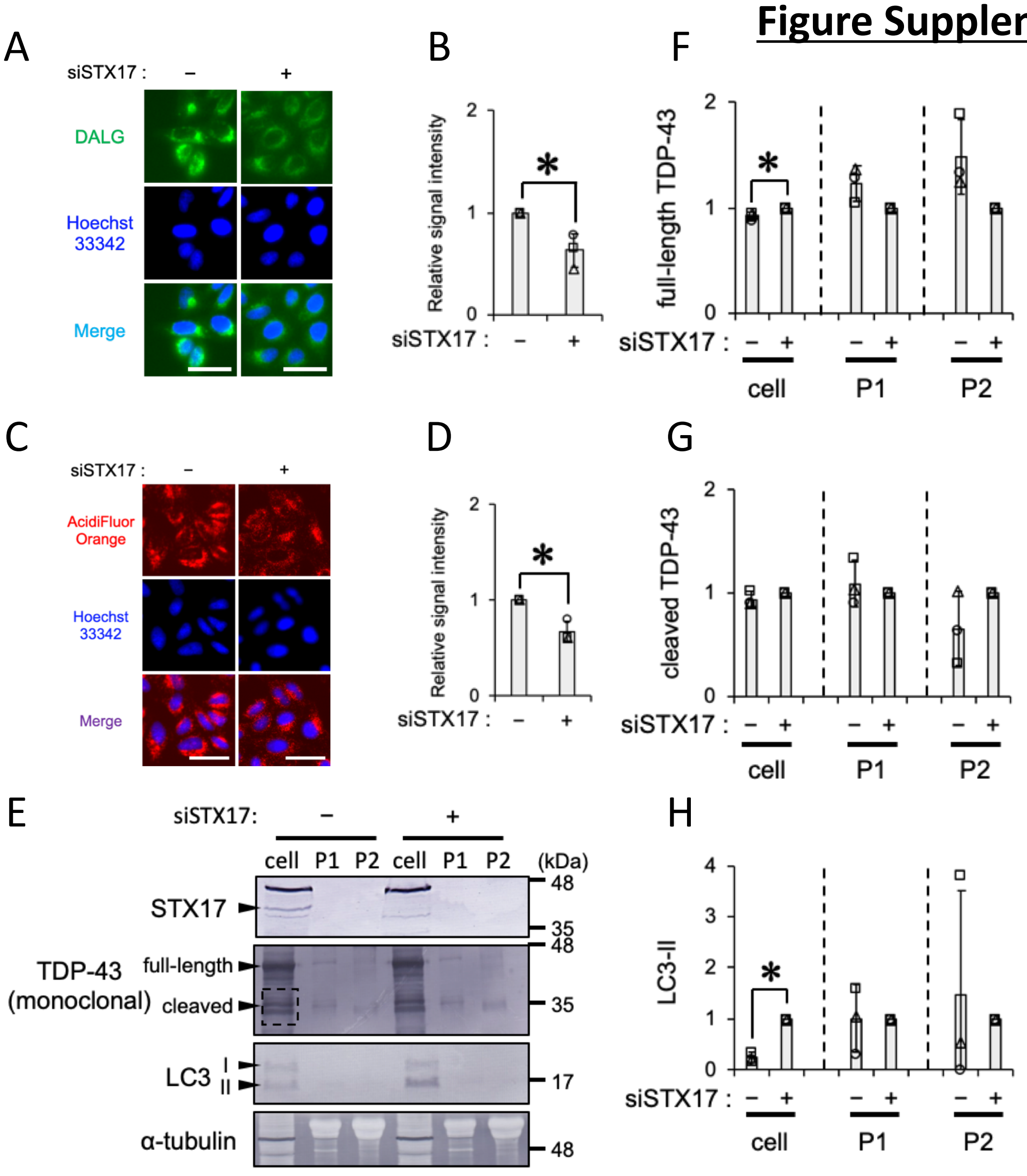

Figure supplement 10. The knockdown of *STX17* does not increase the secretion of TDP-43 or LC3-II

(**A-B**) HeLa cells transfected with control or *STX17* siRNA for 48 hrs were stained with DALGreen and incubated for 2 hrs. (**A**) Representative live-cell images stained with DALGreen (Green) are shown. Nuclei were stained with Hoechst33342 (Blue). Scale bar, 50 µm. (**B**) The relative signal intensity normalized against cells transfected with control siRNA were calculated from three independent experiments including at least 153 cells. (**C-D**) HeLa cells transfected with control or *STX17* siRNA for 48 hrs were stained with AcidiFluor Orange, and the acidity of lysosomes was compared. (**C**) Representative live-cell images stained with AcidiFluor Orange are shown. Nuclei were stained with Hoechst33342 (Blue). Scale bar: 50 µm. (**D**) The relative signal intensity normalized against cells transfected with control siRNA were calculated from three independent experiments including at least 335 cells. (**E-H**) HeLa cells transfected with control or *STX17* siRNA were incubated for 72 hrs. The cell, P1, and P2 fractions were prepared as shown in the Experimental Procedures. (**E**) Representative immunoblots (STX17, TDP-43, LC3, and α-tubulin) in the cell, P1, and P2 fractions are shown. The dashed square indicates the bands of cleaved TDP-43 used for quantification. (**F-H**) Densitometric data on (**F**) full-length TDP-43, (**G**) cleaved TDP-43, and (**H**) LC3-II were calculated from immunoblotting results. The relative signal intensity normalized against cells transfected with *STX17* siRNA are shown. In bar graphs, data are presented as means ± S.D. ($N = 3$). * indicates P <0.05 by the two-tailed unpaired *t*-test.

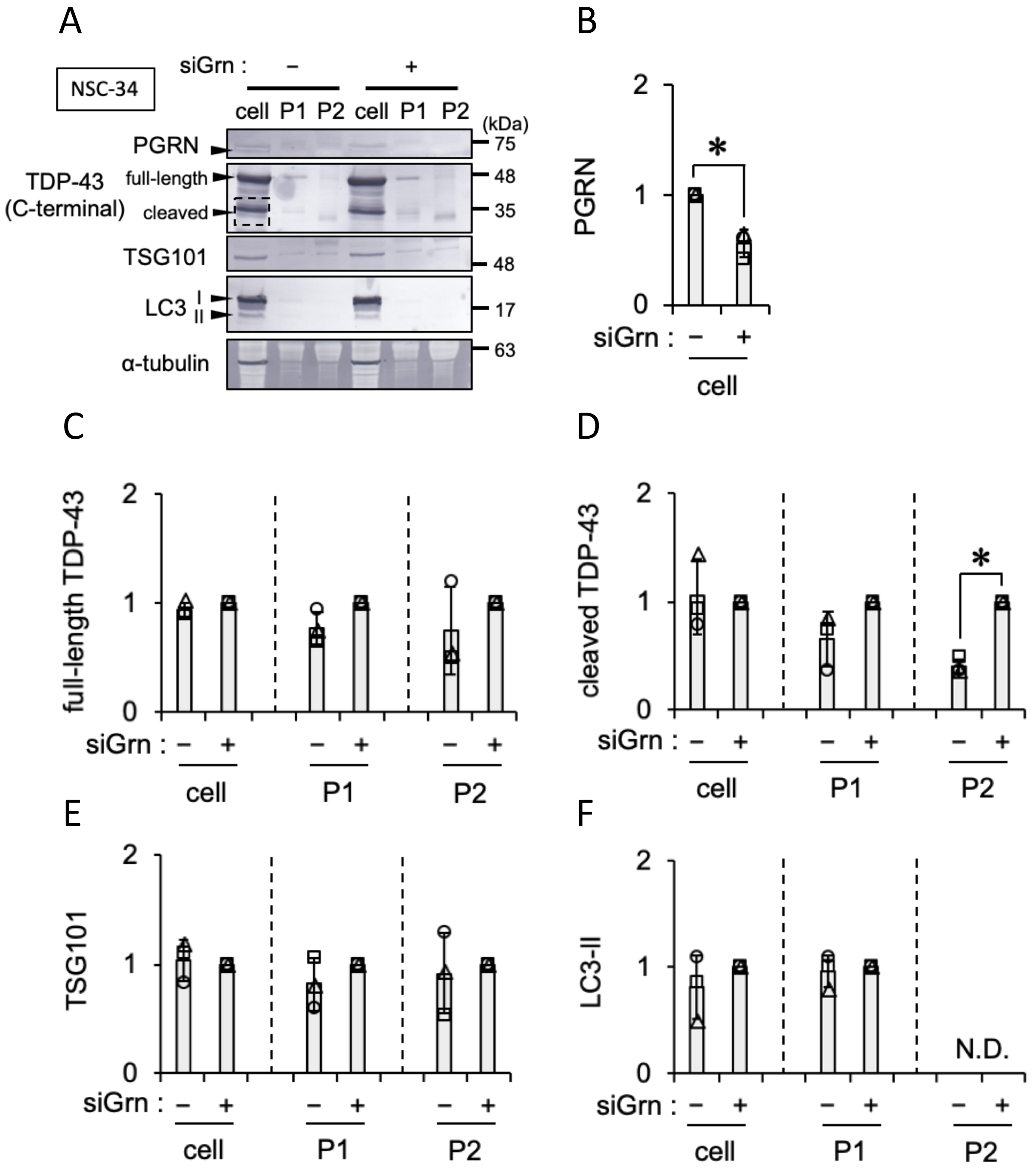

figure supplement 11. The knockdown of *Grn* increases the secretion of cleaved TDP-43 in NSC-34 cells.

NSC-34 cells were transfected with control or *Grn* siRNA for 72 hrs. The cell, P1, and P2 fractions were prepared as shown in the Experimental Procedures. (**A**) Representative immunoblots (PGRN, TDP-43, TSG101, LC3, and α-tubulin) in the cell, P1, and P2 fractions are shown. The dashed square indicates bands of cleaved TDP-43 used for quantification. **(B-F)** Densitometric data on (**B**) PGRN, (**C**) full-length TDP-43, **(D)** cleaved TDP-43, (**E**) TSG101, and (**F**) LC3-II were calculated from immunoblotting results. The relative signal intensity normalized against cells transfected with control or *Grn* siRNA are shown. In bar graphs, data are presented as means ± S.D. ($N = 3$). * indicates $P < 0.05$ by the two-tailed unpaired *t*-test.
